# Overcoming denominator problems in refugee settings with fragmented electronic records for health and immigration data: a prediction-based approach

**DOI:** 10.1186/s12874-024-02204-7

**Published:** 2024-04-01

**Authors:** Stella Erdmann, Rosa Jahn, Sven Rohleder, Kayvan Bozorgmehr

**Affiliations:** 1 Institute of Medical Biometry, University of Heidelberg, Im Neuenheimer Feld 130.3, 69120 Heidelberg, Germany; 2 Section Health Equity Studies and Migration, Department of General Practice and Health Services Research, Heidelberg University Hospital, 69120 Heidelberg, Germany; 3 Department of Population Medicine and Health Services Research, School of Public Health, Bielefeld University, 33501 Bielefeld, Germany

**Keywords:** Asylum seekers, Refugees, Prediction, Generalized linear models, Disease prevalence, Patient-registries

## Abstract

**Background:**

Epidemiological studies in refugee settings are often challenged by the denominator problem, i.e. lack of population at risk data. We develop an empirical approach to address this problem by assessing relationships between occupancy data in refugee centres, number of refugee patients in walk-in clinics, and diseases of the digestive system.

**Methods:**

Individual-level patient data from a primary care surveillance system (PriCare*net*) was matched with occupancy data retrieved from immigration authorities. The three relationships were analysed using regression models, considering age, sex, and type of centre. Then predictions for the respective data category not available in each of the relationships were made. Twenty-one German on-site health care facilities in state-level registration and reception centres participated in the study, covering the time period from November 2017 to July 2021.

**Results:**

445 observations (“centre-months”) for patient data from electronic health records (EHR, 230 mean walk-in clinics visiting refugee patients per month and centre; standard deviation sd: 202) of a total of 47.617 refugee patients were available, 215 for occupancy data (OCC, mean occupancy of 348 residents, sd: 287), 147 for both (matched), leaving 270 observations without occupancy (EHR-unmatched) and 40 without patient data (OCC-unmatched). The incidence of diseases of the digestive system, using patients as denominators in the different sub-data sets were 9.2% (sd: 5.9) in EHR, 8.8% (sd: 5.1) when matched, 9.6% (sd: 6.4) in EHR- and 12% (sd 2.9) in OCC-unmatched. Using the available or predicted occupancy as denominator yielded average incidence estimates (per centre and month) of 4.7% (sd: 3.2) in matched data, 4.8% (sd: 3.3) in EHR- and 7.4% (sd: 2.7) in OCC-unmatched.

**Conclusions:**

By modelling the ratio between patient and occupancy numbers in refugee centres depending on sex and age, as well as on the total number of patients or occupancy, the denominator problem in health monitoring systems could be mitigated. The approach helped to estimate the missing component of the denominator, and to compare disease frequency across time and refugee centres more accurately using an empirically grounded prediction of disease frequency based on demographic and centre typology. This avoided over-estimation of disease frequency as opposed to the use of patients as denominators.

**Supplementary Information:**

The online version contains supplementary material available at 10.1186/s12874-024-02204-7.

## Background

Epidemiological studies of disease prevalence or incidence in refugee settings are often challenged by a lack of denominator data accurately capturing the population at risk. Relating case numbers of a given outcome within a defined period of time to a clearly defined denominator is a fundamental prerequisite for comparisons of disease frequency across time (e.g. comparing change in disease patterns) or space (e.g. different patterns in different settings). This “denominator problem” is well known in health services research, especially in the field of primary care in countries lacking patient-registries or fixed catchment areas for primary care practices [1–4]. Similar challenges occur in population-based studies, where outcomes have been measured but data on the population at risk is not readily available [5, 6] leading to potential bias in disease estimates, especially when comparing disease burden across time and space. Also, rates of health care utilisation cannot be calculated as it is only known how many persons used health services, but not how many actually did not [1, 2].

This is all the more relevant in the context of refugee migration, where the population at risk is characterised by high mobility, and where settings for epidemiological studies are usually camps or camp-like accommodation centres with high fluctuation and population dynamics. Knowledge of disease frequency is very important in these settings for monitoring health needs and planning health services, but also for surveillance and early detection of transmission of infectious diseases [7]. Refugee camps or camp-like accommodation centres (hereafter referred to as “refugee centres”) may offer on-site primary care structures or walk-in clinics [8] where patients are treated and medical diagnoses are captured in electronic health records (EHR). Such data is often used for a plethora of studies [9]. However, the population at risk is usually the total number of inhabitants of the refugee centre, i.e. the occupancy, not the number of patients who utilised the health services. Such data is, however, not always recorded [10].

If occupancy data is recorded by immigration authorities in charge of the refugee centre, it is usually kept in records which are not accessible to health authorities or health professionals who mandate the EHR. The fragmented data ecosystem [10, 11] impedes health monitoring and leads to a situation in which denominator data are practically not available for epidemiological studies in refugee settings. Data protection laws, e.g. under the General Data Protection Regulation of the European Union, impede simplistic linkage of the distinct records into a centralised database. Overcoming the divide between occupancy data in refugee centres and health services utilisation and patient data in a privacy-preserving way is desirable and paramount for meaningful epidemiological research and continuous health monitoring. Precise and reliable knowledge of the relationship between occupancy data and health services utilisation and disease frequency can inform refugee settings in two ways. First, it may help to predict denominator data in settings where no information on occupancies is available. Second, where no functional EHR is in place, denominator data can be used to predict expected rates of disease frequency to inform health services planning. Such knowledge, however, needs to be based on an empirical, reproducible and reliable basis.

The aim of this study is to develop and test an empirical approach addressing the denominator problem in refugee settings, where (a) health *and* occupancy data are available, but recorded within a fragmented data ecosystem (i.e. data are kept in separate records which preclude simplistic linkage), or (b) only *one* of the above data categories is available while the others are missing or not accessible (i.e., the number of patients with a given condition in a defined period of time is available but no occupancy data is given, or vice versa).

To this end, we use the example of Germany and a unique longitudinal data source to assess the relationship between occupancy data in refugee centres, number of patients in on-site walk-in clinics, and selected health outcomes (diseases of the digestive system), respectively. Based on these relationships, we make predictions for refugee centres in which one of these data categories is not available.

## Methods

The overall methodological approach consists of three steps, which are described in detail in the following sections. Firstly, we used individual-level patient data from a primary care surveillance system (see Sect. 2.1) and matched these with aggregated (age- and sex stratified) occupancy data retrieved from authorities per month and centre (see Sect. 2.2). The matched data was then used in a second step to analyse three distinct relationships between occupancy data in refugee centres, number of patients in on-site walk-in clinics, and selected health outcomes:

1. Relationship 1 “disease incidence”: the incidence of diseases of the digestive system in a centre per month, depending on the proportion of male patients, the proportion of adult patients, the total number of patients (per month and centre), and the type of centre.
2. Relationship 2 “patient-occupancy ratio when occupancy data is given”: the number of patients in a centre per month depending on the proportion of male occupancy, the proportion of adult occupancy, the total occupancy (per month and centre), and the type of centre.
3. Relationship 3 “patient-occupancy ratio when patient data is given”: the occupancy number in a centre per month centre depending on the proportion of male patients, adult patients, and the total number of patients (per month and centre), and the type of centre.

The analysis was performed by fitting three models (one for each of the relationships 1–3) and to make predictions for the respective data category not available in each of the relationships 1–3 (compare Sect. 2.3) in a final step.

### Setting and data sources

The present analysis is set in the health monitoring network PriCare*net* [12]. The network is led by the University Hospital Heidelberg and consists of health care providers running on-site health care facilities (as of March 2022) at a total of 25 state-level registration (REG) and reception centres (REC) and 3 district-level accommodation centres for refugees in Germany. Through the network, health care providers are supplied with a tailored EHR (Refugee Care Manager, RefCare©), which, in addition to all features of medical record-keeping, includes a built-in surveillance module. The surveillance module can be activated by authorised personnel at the health care facilities and performs an automated analysis of the locally stored medical routine data based on pre-defined indicators which are operationalised by means of a harmonised analysis script that is identical across all facilities.

The analysis generates anonymous count and incidence data for a total of 65 health care and health service indicators. All observations < 3 are set to 0 to maintain anonymisation and the anonymous monitoring results are exported to the research team at Heidelberg University Hospital for further analysis. The routine local analysis can be run on a monthly basis. Details of the surveillance infrastructure, the monitoring network and the local analysis of indicators are reported elsewhere [12, 13].

A total of 21 on-site health care facilities in state-level registration centres (REG) and reception centres (REC) in Germany participated in the analysis and exported monitoring results to the research team (four did not participate, no reasons given). These centres are categorised by their location within the asylum system: REG, where individuals seeking international protection are first registered and accommodated, and REC which subsequently receive and accommodate individuals until they are transferred to accommodation centres at the district level. REG are characterised by a higher number of refugees and a shorter duration of stay (from a few days to a few weeks), while REC generally accommodate a lower number of refugees for a longer period of time. Of the 21 centres included in the present analysis, 5 are REG and 16 are REC. The 21 centres are located in the German states of Baden-Wuerttemberg, Bavaria, and Hamburg. These states together receive about 30% of the asylum-seeking population in Germany based on administrative quota [14].

The data presented in this paper covers the time period from November 2017 to July 2021. The 21 included facilities had all enrolled into the surveillance network at different points in time and due to closure or changes in health care providers, five centres have since left the network but provided their anonymous surveillance for the purpose of this study. The time in which refugee centres contributed their data to the surveillance network therefore ranges from two to 45 months per centre.

### Health and socio-demographic patient-level data from the EHR

Using the 21 refugee centres and months as units of analysis, the dataset includes 445 observations (i.e. 445 “centre-months”) of recorded medical data with an average of ${mean(n}_{pat})=230$ (standard deviation $sd\left({n}_{pat}\right)=202$) refugee patients visiting walk-in clinics each month and centre. The sample comprised 102.343 of such visits (= $\sum _{i=1}^{445}{n}_{pat}^{i}$, where ${n}_{pat}^{i}$ is the number of refugee patients of “centre-month” $i$) of a total of 47.617 refugee patients. For these 445 centre-months, we have access to reported monitoring data on the number of male, female, adult (≥18 years of age), underage (<18 years of age) patients and total number of patients; as well as data on the incidence of diseases of the digestive tract (based on ICD-10 Codes K00-K95) by centre and month. Diseases of the digestive tract was chosen as relevant ICD category as it is a frequently coded category which contains diseases with relevance for populations in crowded conditions, is sensitive to temporal and seasonal changes and demographics of the population, and reflects not only somatic conditions but also psychosomatic aspects associated with the stressful conditions of living in refugee centre. Furthermore, we have access to the countries of origin of the patients. In a sensitivity analysis, we included information of the country of origin in the analysis (see Appendix A.3.3).

### Occupancy data and aggregate-level socio-demographics

In addition, we collected data on the occupancy of each of the refugee centres represented in the PriCare*net* surveillance network through a monthly online-survey among authorities in charge of the centre. The census survey was initiated in October 2018 and includes count data on the number of adults (18 years or above) and children (below 18 years) by sex (male/female), respectively, living in the given centre on the 15th of the respective month. Participation in this survey is voluntary and we were able to collect occupancy data from 6 REG and 13 REC resulting in a total of 215 centre-months (October 2018 until and including October 2021). The mean occupancy is ${mean(n}_{occ})=348$ with $sd\left({n}_{occ}\right)=287$per centre and month.

The total occupancy of each centre and month for all adults was calculated by adding up the reported numbers of male and female adults and for children, respectively. The total numbers of children and adults were then added to generate the total occupancy of each centre for each of the 215 centre-months. This left a total of 52.7% of the centre-months prone to the ‘denominator problem’, i.e. without information on occupancy as population at risk for the calculation of disease frequencies beyond the number of patients.

### Description of derived datasets

In order to address relationship 1 (disease incidence), we used the EHR data. For modelling the relationships 2 and 3, i.e., addressing the “denominator problem”, we matched the EHR data with the occupancy data for each month and centre where available (matched data). For 33 observations there was more than one report of the occupancy for one centre in one month, in these cases the mean number of patients (according to sex- and age-strata) was taken and rounded to a natural number. The unmatched EHR (EHR unmatched) and occupancy data (occupancy unmatched) were then used to make predictions based on the models fitted for modelling relationships 3 and 2, respectively.

Observations of the matched data set for which the occupancy number is smaller than the number of patients (i.e. ${\text{n}}_{\text{o}\text{c}\text{c}\text{u}\text{p}}<{\text{n}}_{\text{p}\text{a}\text{t}}$) were excluded from the analysis. This pattern is plausible when there is a high turn-around in centres, i.e. a high volume of incomings and transfers, with individuals who utilise health services on-site, but stay in the centres only for a short time. However, for sensitivity checks, the models of Sect. 2.3.2 and 2.3.3 were also fitted on the complete matched data set (compare Appendix A.3.1) which included also such observations.

Another sensitivity analysis concerned the data processing due to data protection issues: as data of the EHR with less than 3 observations in one strata are set to 0 by default, it cannot be distinguished in the analysed data if a 0 count is a “true” 0, 1 or 2. As diseases of the digestive system are not rare, one could argue, that 0 cases in one month are unrealistic and probably rather due to lack of reporting, i.e., missing information, than due to the anonymisation process. Therefore, we conducted a sensitivity analysis in which we excluded observations with 0 cases of diseases of the digestive system (compare Appendix A.3.2).

In another sensitivity analysis, we included information of the country of origin in the analysis (see Appendix A.3.3).

### Description of regression models

In the following, the regression models used to describe relationships 1–3 are presented. The results of the fitted models can be found in Sect. 3.2.

The analyses were conducted in R version 4.2.1 using packages glmmTMB for fitting mixed-effects models [15] and DHARMa for performing model diagnostics [16]. The original R output can be found in Appendix A.2.

#### Negative binominal model (relationship 1: disease incidence)

Relationship 1, i.e., the association between the number of cases of diseases of the digestive system and the number of patients and the percentage of male and adult patients is modelled by a negative binominal model with first-order autoregressive process, including a zero-inflation model and a dispersion model fitted on the EHR data. The model allows the conditional mean to depend on the percentage of adult (adult) and male (male) patients, as well as on the number of patients (${\text{n}}_{\text{p}\text{a}\text{t}}$). The model assumes structural zeros to depend on the number of patients and the dispersion parameter to depend on the type of centre (see R-syntax in appendix A.2.1). As the variables percentage of male and female, as well as the variables percentage of adult and underage patients are correlated, we chose (without loss of generality) one variable of each. We chose to include the variable number of patients into the conditional model, as the model including this variable had a smaller AIC as compared to the respective model containing the variable type of centre or containing both variables. In the zero-inflation part of this model we modelled the intercept in order to present the baseline odds for being among the centres who never code cases of diseases of the digestive system. Furthermore, we adjusted for the number of patients per centre and month, as we think that especially centres with a low number of patients are prone to underreporting of cases of diseases of the digestive system in our study. We also checked the variable type of centre instead, but the AIC of this model was larger. In the dispersion model, the natural choice for the covariate was type of centre (see sd and Q1-Q3 of Incidence of diseases of the digestive system with respect to patients in Table A.1). Alternatively, we checked the variable number of patients, but the respective model had a larger AIC. Thus, the model can be represented by the following set of equations:

$${\mu }=\text{E}\left(\text{c}\text{o}\text{u}\text{n}\text{t}|\text{N}\text{S}\text{Z}\right)=\text{exp}\left({{\beta }}_{0}+{{\beta }}_{\text{a}\text{d}\text{u}\text{l}\text{t}}+{{\beta }}_{\text{m}\text{a}\text{l}\text{e}}+{{\beta }}_{{\text{n}}_{\text{p}\text{a}\text{t}}}\right),$$

$${{\sigma }}^{2}=\text{V}\text{a}\text{r}\left(\text{c}\text{o}\text{u}\text{n}\text{t}|\text{N}\text{S}\text{Z}\right)= {\mu }\left(1+\frac{{\mu }}{{\theta }}\right),$$

$$\text{l}\text{o}\text{g}\text{i}\text{t}\left(\text{p}\right) = {{\beta }}_{0}^{\left(\text{z}\text{i}\right)}+{{\beta }}_{{\text{n}}_{\text{p}\text{a}\text{t}}}^{\left(\text{z}\text{i}\right)},$$

$$\text{l}\text{o}\text{g}\left({\theta }\right)={{\beta }}_{0}^{\left(\text{d}\text{i}\text{s}\text{p}\text{e}\text{r}\text{s}\text{i}\text{o}\text{n}\right)} +{{\beta }}_{\text{t}\text{y}\text{p}\text{e} \text{o}\text{f} \text{c}\text{e}\text{n}\text{t}\text{r}\text{e}}^{\left(\text{d}\text{i}\text{s}\text{p}\text{e}\text{r}\text{s}\text{i}\text{o}\text{n}\right)}\cdot \text{t}\text{y}\text{p}\text{e}\ \text{o}\text{f}\ \text{c}\text{e}\text{n}\text{t}\text{r}\text{e},$$

where $\text{N}\text{S}\text{Z}$ is the event “non-structural zero”, $\text{p}=1-\text{P}\text{r}\left(\text{N}\text{S}\text{Z}\right)$ is the zero-inflation probability and ${\beta }$’s are the regression coefficients with subscript denoting the covariate and with 0 denoting the intercept (compare [15]). The parameterization of the negative binomial is chosen as family = nbinom2 (compare Appendix A.2.1). Therefore, the variance increases quadratically with the mean as ${{\sigma }}^{2}= {\mu }(1 + {\mu }/{\theta })$, with ${\theta }>0$ [17]. Furthermore, an *AR(1)* covariance structure is used to model a first-order autocorrelation for consecutive months $t=\left\{1,\dots ,N\right\}$ (i.e. the $\text{X}\left(\text{t}\right)$ stationary *AR(1)* process has covariance $\text{c}\text{o}\text{v}(\text{X}\left(\text{t}\right), \text{X}(\text{s}\left)\right)={{\sigma }}^{2}\text{e}\text{x}\text{p}(-{\theta }|\text{t}-\text{s}\left|\right)$).

#### Generalized linear model (relationship 2: patient-occupancy ratio when occupancy data is given)

In order to model the number of patients depending on the percentage of male and adult occupancy and the type of centre (REC or REG), a Gaussian model was fitted on the matched data for the continuous outcome variable $\text{r}={\text{n}}_{\text{p}\text{a}\text{t}}/{\text{n}}_{\text{o}\text{c}\text{c}\text{u}\text{p}}$. Therefore, the ratio $\text{r}$ was calculated based on the number of patients (${\text{n}}_{\text{p}\text{a}\text{t}}$) and the number of persons living in a centre per month (${\text{n}}_{\text{o}\text{c}\text{c}\text{u}\text{p}}$). As the percentage of male and female, as well as the percentage of adult and underage occupancy is correlated, we chose one variable of each, namely male and adult. Inclusion of type of centre and occupancy number into the model resulted in the smallest AIC (compared to including just one of both). Not including the covariate type of centre or occupancy number in the dispersion model resulted in a lower AIC. Therefore, these covariates were not included in the dispersion model.

#### Generalized linear model (relationship 3: patient-occupancy ratio when patient data is given)

To model the occupancy number depending on the percentage of male and adult patients and the type of centre, a Gaussian model was fitted on the matched data for dependent variable $\text{r}$ (see 2.3.2 above) while accounting for overdispersion including number of patients into the dispersion model (which showed the smallest AIC compared to including type of centre instead or both).

Model fit with respect to model diagnostics was performed by means of qq-plots to detect overall deviations from the expected distribution, tests for correct distribution (KS test), dispersion and outliers (compare Appendix A.2 for details).

## Results

In this section, a description of the data used for fitting the models introduced in Sect. 2.3 (compare Sect. 3.1) and the fitted models itself (compare Sect. 3.2) are presented. Furthermore, the predictions made for the unmatched data sets based on the models are presented (compare Sect. 3.3).

### Description of the data

Table 1 shows a description of the data used in this study, i.e. the EHR data, the occupancy data, the matched data, the unmatched EHR data and the unmatched occupancy data. A more detailed description with respect to the distribution of the type of centres (REC/ REG) can be found in Appendix A.1 (compare Tables A1, A2, A3, A4 and A5).

**Table 1:** Description of data. Absolute and relative frequencies and mean, standard deviation, median and interquartile range (Q1 - Q3), minimum and maximum of the observed/predicted variables of the EHR data, occupancy data, matched, unmatched EHR and unmatched occupancy data sets are given, respectively. In case the predicted variables are described, boxes indicate the model and data set used (– - – - –: predictions made on the basis of model of Sect. 2.3.2 and 2.3.1 modelling relationships 2 (patient-occupancy ratio when occupancy data is given) and 1 (disease incidence); -----: predictions made on the basis of model of Sect. 2.3.2 modelling relationship 2 (patient-occupancy ratio when occupancy data is given); $\cdot\cdot\cdot\cdot\cdot\cdot\cdot$: predictions made on the basis of model of Sect. 2.3.3 modelling relationship 3 (patient-occupancy ratio when patient data is given)). In the Tables of Appendix A.1, the description is done stratified for the type of centre

Variables	EHR	occupancy data	matched	EHR unmatched	occupancy unmatched
	(*N* = 445)	(*N* = 215)	(*N* = 147)	(*N* = 270)	(*N* = 40)
**Centre**					
1	22 (5%)	5 (2%)	3 (2%)	19 (7%)	2 (5%)
2	6 (1%)	8 (4%)	2 (1%)	1 (0%)	3 (8%)
3	0 (0%)	1 (0%)	0 (0%)	0 (0%)	1 (2%)
4	37 (8%)	17 (8%)	11 (7%)	21 (8%)	1 (2%)
5	22 (5%)	13 (6%)	13 (9%)	9 (3%)	0 (0%)
6	32 (7%)	19 (9%)	15 (10%)	15 (6%)	2 (5%)
7	38 (9%)	33 (15%)	27 (18%)	8 (3%)	3 (8%)
8	7 (2%)	1 (0%)	1 (1%)	6 (2%)	0 (0%)
9	23 (5%)	14 (7%)	5 (3%)	10 (4%)	1 (2%)
10	19 (4%)	14 (7%)	7 (5%)	12 (4%)	7 (18%)
11	27 (6%)	14 (7%)	13 (9%)	14 (5%)	1 (2%)
12	16 (4%)	0 (0%)	0 (0%)	16 (6%)	0 (0%)
13	27 (6%)	14 (7%)	9 (6%)	16 (6%)	3 (8%)
14	16 (4%)	17 (8%)	9 (6%)	7 (3%)	8 (20%)
15	17 (4%)	12 (6%)	11 (7%)	6 (2%)	1 (2%)
16	0 (0%)	1 (0%)	0 (0%)	0 (0%)	1 (2%)
17	25 (6%)	18 (8%)	16 (11%)	9 (3%)	2 (5%)
18	16 (4%)	4 (2%)	4 (3%)	12 (4%)	0 (0%)
19	22 (5%)	2 (1%)	1 (1%)	21 (8%)	1 (2%)
20	44 (10%)	0 (0%)	0 (0%)	44 (16%)	0 (0%)
21	6 (1%)	8 (4%)	0 (0%)	1 (0%)	3 (8%)
22	5 (1%)	0 (0%)	0 (0%)	5 (2%)	0 (0%)
23	18 (4%)	0 (0%)	0 (0%)	18 (7%)	0 (0%)
**type of centre**					
REC	345 (78%)	137 (64%)	104 (71%)	234 (87%)	26 (65%)
REG	100 (22%)	78 (36%)	43 (29%)	36 (13%)	14 (35%)
**Male**					
N	445	215	147	270	40
mean	60	66	60	59	69
sd	14	14	13	14	17
median	60	65	58	60	65
Q1 - Q3	54–68	59–74	52–69	54–67	61–72
min - max	0–100	24–98	23–90	0–100	24–98
**Adult**					
N	445	215	147	270	40
mean	80	77	80	80	78
sd	10	10	10	11	13
median	81	77	81	81	75
Q1 - Q3	74–87	70–84	72–87	75–86	69–86
min - max	0–100	54–114	48–100	0–100	58–100
**diseases of the digestive system**					|– - – - – - – - – - – -
N	445	0	147	270	24
Nmiss	0 (0%)	215 (100%)	0 (0%)	0 (0%)	16 (40%)
mean	23	NA	21	23	17
sd	23	NA	23	24	7.2
median	12	NA	11	12	15
Q1 - Q3	5–35	NA -- NA	5–31	5–37	12–19
min - max	0–89	NA -- NA	0–83	0–89	6.6–40
**n_pat**					– - – - – -|/|------------
N	445	0	147	270	40
Nmiss	0 (0%)	215 (100%)	0 (0%)	0 (0%)	0 (0%)
mean	230	NA	212	233	153
sd	202	NA	195	208	121
median	149	NA	127	150	118
Q1 - Q3	93–304	NA -- NA	95–227	85–316	95–169
min - max	5–934	NA -- NA	29–934	5–934	25–552
**n_occup**				|·················	-----------------------|
N	0	215	147	270	40
Nmiss	445 (100%)	0 (0%)	0 (0%)	0 (0%)	0 (0%)
mean	NA	348	394	448	263
sd	NA	287	312	394	209
median	NA	244	266	286	210
Q1 - Q3	NA -- NA	164–449	193–503	182–583	130–296
min - max	NA -- NA	32–1516	32–1516	14–1821	56–958
**Incidence of diseases of the digestive system with respect to patients**				·················|	|– - – - – - – - – - – -
N	445	0	147	270	24
Nmiss	0 (0%)	215 (100%)	0 (0%)	0 (0%)	16 (40%)
mean	0.092	NA	0.088	0.096	0.12
sd	0.059	NA	0.051	0.064	0.029
median	0.092	NA	0.094	0.092	0.12
Q1 - Q3	0.056–0.12	NA -- NA	0.054–0.12	0.057–0.13	0.099–0.14
min - max	0–0.3	NA -- NA	0–0.23	0–0.3	0.081–0.19
**Incidence of diseases of the digestive system with respect to occupancy**				|·················	– - – - – - – - – - – -
N	0	0	147	270	24
Nmiss	445 (100%)	215 (100%)	0 (0%)	0 (0%)	16 (40%)
mean	NA	NA	0.047	0.048	0.074
sd	NA	NA	0.032	0.033	0.027
median	NA	NA	0.043	0.045	0.073
Q1 - Q3	NA -- NA	NA -- NA	0.023–0.069	0.027–0.068	0.051–0.091
min - max	NA -- NA	NA -- NA	0–0.14	0–0.16	0.037–0.13
**ratio = n_pat/n_occup**				·················	– - – - – -|/|------------
N	0	0	147	270	40
Nmiss	445 (100%)	215 (100%)	0 (0%)	0 (0%)	0 (0%)
mean	NA	NA	0.54	0.51	0.6
sd	NA	NA	0.19	0.083	0.14
median	NA	NA	0.5	0.5	0.59
Q1 - Q3	NA -- NA	NA -- NA	0.39–0.69	0.47–0.53	0.49–0.67
min - max	NA -- NA	NA -- NA	0.13–0.99	0.23–0.77	0.38–0.91
				·················	|-----------------------|

### Results of the fitted models

#### Negative binominal model (relationship 1: disease incidence)

The fixed effect results of the negative binominal model with first-order autoregressive process and zero-inflation as well as dispersion model can be found in Table 2. It was fitted on the EHR data (*n* = 445) and models the relationship of cases of diseases of the digestive system per month and centre depending on the proportion of males and adults, as well as on the number of patients. The interpretation of the results is as follows:

Conditional model: The “baseline” average number of incident cases of diseases of the digestive system is 3.55 (CI: 2.00- 6.30) among all centres who had ever coded a case of diseases of the digestive systems. If the percentage of adult or male patients at a centre increases by 10 units (i.e. 10%-points), the incidence rate of diseases of the digestive system would be expected to increase by a factor of 1.03 (0.96–1.11) and 1.12 (1.05–1.20), respectively, while holding all other variables in the model constant.

Zero inflation model: The baseline odds of being among the centres who never code cases of diseases of the digestive system is 0.44 (0.26–0.76). Each 10 unit increase in the absolute number of patients of a centre decreases the odds of being among those centres by 0.93 (0.89–0.96).

The expected dispersion model coefficients are 3.11 (2.52–3.83) and 7.05 (3.95–12.56) for the intercept and the type of centre, respectively. The first-order autoregressive coefficient is estimated to be 0.92.

**Table 2:** Results of the negative binominal model fitted on the EHR data (relationship1: disease incidence). Estimates of the fixed effects of the conditional, zero-inflation and dispersion model are given with associated 95% confidence intervals (CI). The number of observations is 445, the Akaike information criterion (AIC) is given by 3143.9 and the first-order autoregressive coefficient is given by 0.92. The original R function call and output, as well as model diagnostics can be found in Appendix A.2.1

Model	Variable	incidence rate ratio (IRR)	Lower 95% CI	Upper 95% CI
Conditional	Intercept	3.55	2.00	6.30
	adult (10%)	1.03	0.96	1.11
	male (10%)	1.12	1.05	1.20
	${\text{n}}_{\text{p}\text{a}\text{t}}/10$	1.03	1.03	1.03
Zero-inflation	Intercept	0.44	0.26	0.76
	${\text{n}}_{\text{p}\text{a}\text{t}}/10$	0.93	0.89	0.96
Dispersion	Intercept	3.11	2.52	3.83
	Type of centre, REG (ref: REC)	7.05	3.95	12.56

#### Generalized linear model (relationship 2: patient-occupancy ratio when occupancy data is given)

Table 3 shows the results of the Gaussian model with dispersion model fitted on the matched data. In this model, the relationship between the number of patients (${\text{n}}_{\text{p}\text{a}\text{t}}$) and the occupancy number living in a centre per month (${\text{n}}_{\text{o}\text{c}\text{c}\text{u}\text{p}}$) is modelled by a ratio $\text{r}={\text{n}}_{\text{p}\text{a}\text{t}}/{\text{n}}_{\text{o}\text{c}\text{c}\text{u}\text{p}}$ as dependent variable and using the proportion of male occupancy and adult occupancy, as well as the occupancy number in a centre per month and the type of centre (REG vs. REC) as independent variables. The interpretation is as follows: For every 10 unit increase in the percentage of adults of the occupancy of a centre, the ratio r increases by 0.08 (ceteris paribus). Everything else held equal, the ratio increases by 0.32 for REG centres compared to REC centres. A prediction of the ratio and therefore the number of expected patients in a centre in a month was done on the unmatched occupancy data (compare Sect. 3.3).

**Table 3:** Results of the generalized linear model fitted on the matched data (relationship 2: patient-occupancy ratio when occupancy data is given). Estimates of the fixed effects of the conditional and dispersion model are given with associated 95% confidence intervals (CI). The number of observations is 147, the AIC is given by -141.3 and the dispersion estimate for the gaussian family is given by 0.0206. The original R output as well as model diagnostics can be found in Appendix A.2.2

Model	Variable	Estimate	Lower 95% CI	Upper 95% CI
Conditional	Intercept	-0.01	-0.19	0.20
	adult (10%)	0.08	0.04	0.12
	male (10%)	-0.01	-0.04	0.02
	Type of centre, REG (ref: REC)	0.32	0.26	0.39
	${\text{n}}_{\text{o}\text{c}\text{c}\text{u}\text{p}}/10$	0.00	0.00	0.00

#### Generalized linear model (relationship 3: patient-occupancy ratio when patient data is given)

In order to model and predict the ratio and therefore the total number of persons in a centre in a month on the basis of patient data, a Gaussian model with dispersion model was fitted on the matched data. Independent variables of the model are the proportion of male patients and adult patients, as well as the type of centre (REG vs. REC). The results can be found in Table 4, where the interpretation is as follows: For every 10 unit increase in percentage of male patients in a centre, the ratio increases by 0.04, while the ratio increases by 0.20 for REG centres compared to REC centres (everything else held equal).

**Table 4:** Results of the generalized linear model fitted on the matched data (relationship 3: patient-occupancy ratio when patient data is given). Estimates of the fixed effects of the conditional and dispersion model are given with associated 95% confidence intervals (CI). The number of observations is 147, the AIC is given by -92.6. The original R output as well as model diagnostics can be found in Appendix A.2.3

Model	Variable	Estimate	Lower 95% CI	Upper 95% CI
Conditional	Intercept	0.37	0.14	0.60
	adult (10%)	-0.02	-0.06	0.02
	male (10%)	0.04	0.01	0.07
	Type of centre, REG (ref: REC)	0.20	0.14	0.27
Dispersion	Intercept	-3.35	-3.68	-3.02
	${\text{n}}_{\text{p}\text{a}\text{t}}/10$	-0.01	-0.02	0.00

### Predictions on the basis of the models

**Fig. 1:**
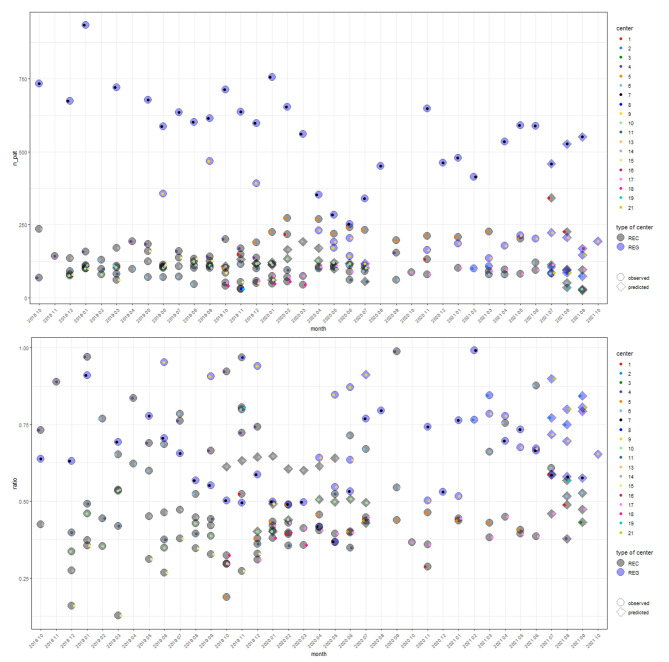
Observations and predictions on relationship 2: patient-occupancy ratio when occupancy data is given. Observed (indicated by circles) and predicted (indicated by diamonds) ratio $\text{r}$ and number of patients ${\text{n}}_{\text{p}\text{a}\text{t}}={\text{n}}_{\text{p}\text{o}\text{p}}\cdot \text{r}$ on the basis of the matched data set and the model of Sect. 2.3.2 for the unmatched occupancy dataset, respectively. Observations/predictions of different facilities are indicated by the colours of the small dots inside the circles/diamonds, which are coloured with respect to the type of centre (REC: black, REG: blue). Analogues figures also indicating 95% confidence intervals for the observed estimates/ predictions can be found in Appendix A.4 (compare Figure A1)

**Fig. 2:**
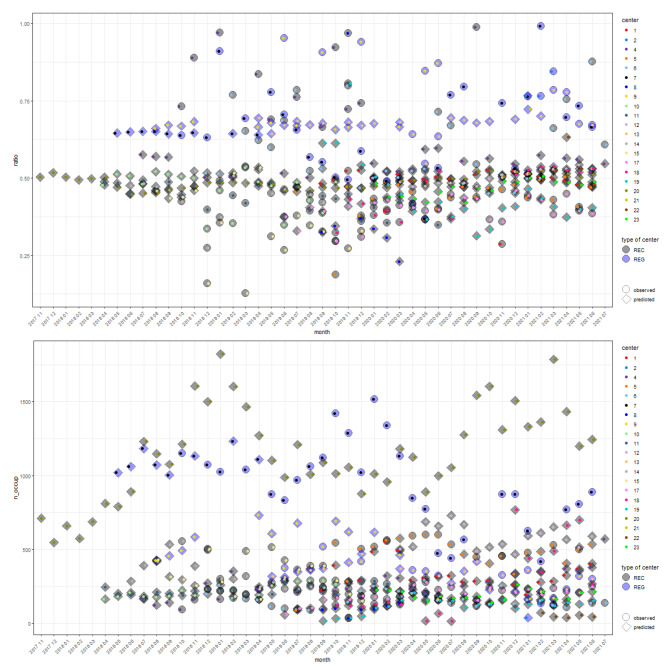
Observations and predictions on relationship 3: patient-occupancy ratio when patient data is given. Observed (indicated by circles) and predicted (indicated by diamonds) ratio $\text{r}$ and occupancy number ${\text{n}}_{\text{o}\text{c}\text{c}\text{u}\text{p}}={\text{n}}_{\text{p}\text{a}\text{t}}/\text{r}$ on the basis of the matched data set and the model of Sect. 2.3.3 for the unmatched EHR dataset, respectively. Observations/predictions of different centres are indicated by the colours of the small dots inside the circles/diamonds, which are coloured with respect to the type of centre (REC: black, REG: blue). Analogous figures with 95% confidence intervals for the observed estimates/predictions can be found in Appendix A.4 (compare Figure A2)

Figures 1 and 2 show the observations as well as the predictions of the ratio and the number of patients and the occupancy number made on the basis of (a) the matched data set and (b) the models presented in Sect. 2.3.2 and 2.3.3 which were applied to the unmatched occupancy and unmatched EHR data sets, respectively.

**Fig. 3:**
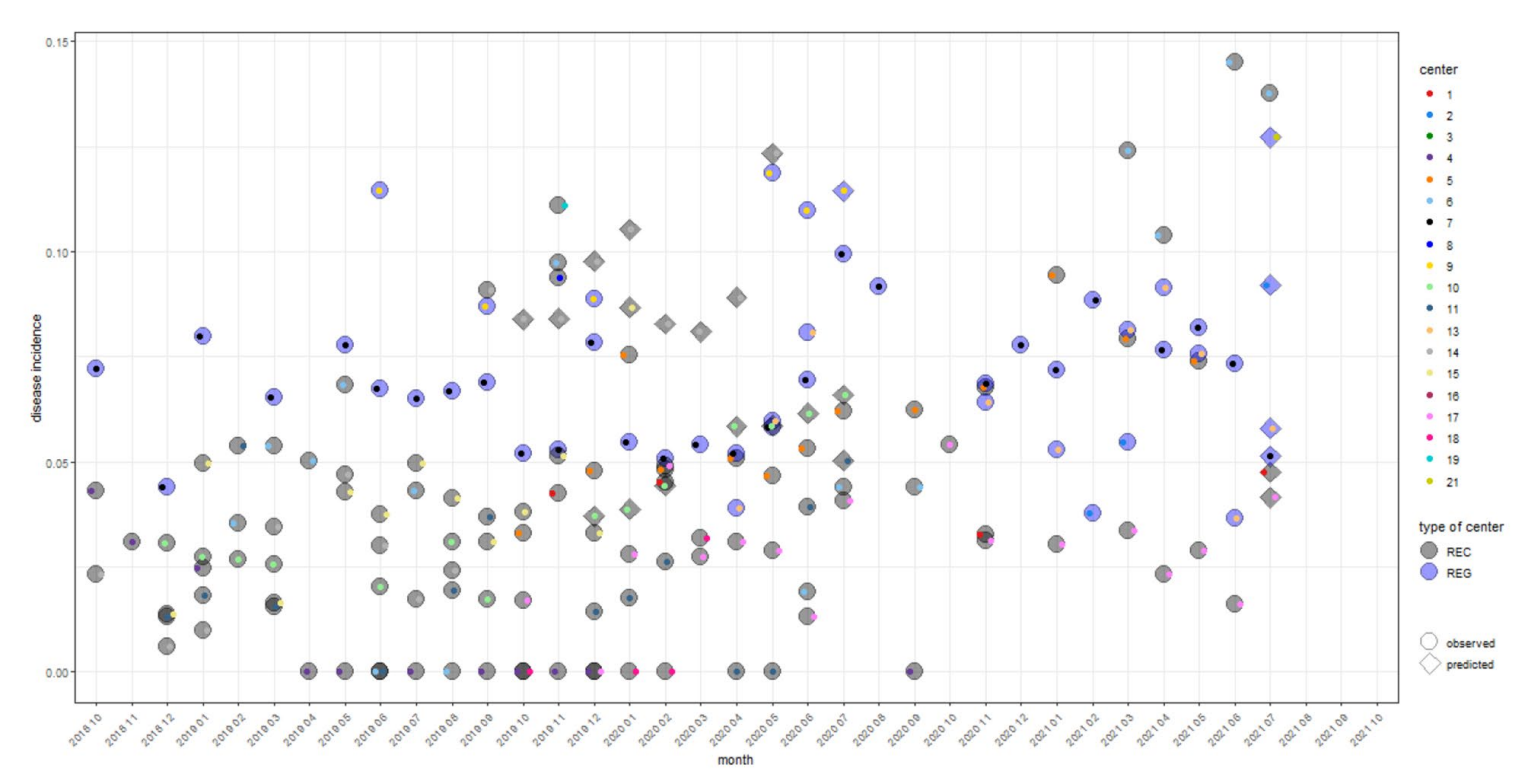
Observations and predictions on relationship 1 and 2: disease incidence depending on total population at risk (occupancy). Observed (indicated by circles) and predicted (indicated by diamonds) disease incidence with respect to occupancy number (i.e., number of incident cases of diseases of the digestive system divided by the occupancy number) on the basis of the EHR data set and the models of Sect. 2.3.2 (relationship 2: patient-occupancy ratio when occupancy data is given) and Sect. 2.3.1 (relationship 1: disease incidence) for the unmatched occupancy dataset, respectively. Observations/predictions of different facilities are indicated by the colours of the small dots inside the circles/diamonds, which are coloured with respect to the type of centre (REC: black, REG: blue). An analogous figure with 95% confidence intervals for the observed estimates/ predictions can be found in Appendix A.4 (compare Figure A3)

In Fig. 3, the results of predicting the number of patients by applying the model of Sect. 2.3.2 to the unmatched occupancy data set first, and then using the predicted number of patients and the same type of centre, male and adult percentage to predict the disease incidence by the model of Sect. 2.3.1 are compared to the observed disease incidence of the matched data set. Uncertainty bounds (95% confidence intervals) for all predictions are presented in the Appendix (Figures A1-A3). The width of the confidence intervals of predicted and observed ratio when occupancy data is available (Figure A1) and when patient data is available (Figure A2) are comparable. The width of the confidence intervals of predicted disease incidence are smaller than the width of the confidence intervals of the observed values (Figure A3).

Results of the sensitivity analysis are presented in Appendix A.3 (Tables A6-A13). Table A6 is a description of the full matched data, i.e., where records for which the occupancy number is smaller than the number of patients (i.e. ${\text{n}}_{\text{o}\text{c}\text{c}\text{u}\text{p}}<{\text{n}}_{\text{p}\text{a}\text{t}}$) were not excluded. Table A7 and A8 show the results of the models described in Sect. 2.3.2 and 2.3.3 fitted on this data set. Overall, the results are similar (compare Appendix A.3.1 for more details). Table A9, A10 and A11 show the results of the models of Sect. 2.3 fitted on the reduced data sets (where we excluded observations with 0 cases of diseases of the digestive system), where the zero-inflation part of the model of Sect. 2.3.1 was dropped. Again, the results of this sensitivity analysis resemble that of the main analysis. Tables A12 and A13 show the results of the models described in Sect. 2.3.1 and 2.3.3 while also including information of the countries of origin of the patients. The effect estimates of these models are comparable to those of the main analyses.

The incidence of diseases of the digestive system, calculated based on the number of patients in different sub-data sets, was found to be 9.2% (sd: 5.9) in the electronic health records (EHR) dataset, 8.8% (sd: 5.1) in the matched dataset, 9.6% (sd: 6.4) in the EHR dataset without matching, and 12% (sd: 2.9) in the unmatched occupancy dataset. When the available or predicted occupancy was used as the denominator, the average incidence estimates (per centre and month) in the matched data were 4.7% (sd: 3.2), 4.8% (sd: 3.3) in the EHR dataset without matching, and 7.4% (sd: 2.7) in the occupancy unmatched dataset (compare Table 1).

## Discussion

Data of the health monitoring network PriCare*net* was affected by the denominator problem, i.e. missing or unavailable data on the population at risk living in the refugee centre. We were able to fit adequate models on the underlying data in order to model the relationship between number of patients and total occupancy with respect to the percentage of males and adults, the number of patients or occupancy (where available) and/or the type of centre. With these models, predictions in both directions are possible, allowing to estimate the missing component provided data on either patients or occupancy is available. The models build on the assumption that the ratio between patients and occupancy for each month can be predicted by information on demography (age, sex) and type of centre. Furthermore, the disease incidence can be predicted based on (estimated) patient information of a centre if only data on total occupancy is available. The predictions made by the models fall into a reasonable range compared to the observed values. In case the disease incidence is predicted on the basis of the occupancy, it is assumed, that the percentage of males and adults is the same among the occupancy and the patients.

The approach has two practical implications for the scientific underpinning of health monitoring systems. Firstly, the frequency of incident diagnoses of diseases can be better compared across time and refugee centres as an estimate of the population at risk was generated, allowing for the calculation of incidence rates with occupancy numbers as denominator (instead of number of patients). This reduces the risk of over- or underestimation of disease frequency. Secondly, the approach allows for an empirically grounded prediction of disease frequency that can be expected based on the occupancy, i.e. the absolute number of refugees living in the centres as well as related age and sex distributions. This allowed us to predict, with reasonable uncertainty, the expected number of diseases of the digestive system per month in refugee centres which did not provide any data on this outcome. Applying this approach to other outcomes, such as cardiovascular diseases, endocrinological disorders, neurological problems, infectious diseases, or mental health conditions could help to inform health services planning in view of migration dynamics and fluctuations of numbers of newly arriving refugees.

The method presented here adds to longstanding challenges of estimating denominators in primary care settings [1, 4] by providing an approach for application in the context of refugee migration. Our approach combines elements previously denoted as “utilization correction factor” approach (which estimates practice denominators from healthcare utilisation rates) [3, 18] or “prediction-based approaches” (which use population demographics as basis for denominator predictions) [1].

However, our approach is limited by important aspects. Morbidity patterns are not only related to age and sex, but may also be related to countries of origin and pre-migration exposures [19, 20], migration routes [21], as well as post-migration contexts [22]. However, the occupancy data obtained from authorities did not provide data on nationalities or county of origin. This lack constitutes a particular problem, as this information can be a relevant exposure or proxy for health risks, and as such be associated with health outcomes. We have conducted a sensitivity analysis incorporating information of the country of origin of the patients into the models. In this sensitivity analysis, we used patient-level information on country of origin to assess potential impacts on the expected cases or denominators. For our outcome (diseases of the digestive system), the results were comparable to those of the main analysis, but this may be different for other outcomes (e.g. infectious diseases), which may show closer relationships between source-country exposures and respective diseases. In these cases, “weights” could be derived from patient-level data based on the approach of our sensitivity analysis and be included in the estimation of the respective relationships of interest. However, these approaches constitute only symptomatic solutions to the more fundamental deficit of non-availability of denominator information in the area of migrant health research, which could be overcome in the future with the implementation and use of appropriate privacy-preserving record linkage strategies to overcome the fragmented the data landscape [23].

Beyond the dichotomous information on types of centres included in the analysis (REG vs. REC) no further information was available on contextual factors that may affect health outcomes among the population (e.g. quality of centre, hygiene, remoteness etc.) [24, 25]. Further information on such data could help to improve predictions, but availability in unified single datasets is unrealistic. Future studies using Bayesian approaches and prior information derived from the empirical literature could help to improve predictions by using more information on contexts than was available in the routine data set. Another limitation relates to data protection, as records of the EHR data with less than 3 observations in one stratum were set to 0. While we could not distinguish if a 0 count is in fact a “true” 0, or rather a “1” or “2”, our sensitivity analysis and the zero-inflated models helped to minimise the problems entailed by anonymisation requirements.

In the refugee centre, occupancy data was collected in a census approach using the mid-month as a cut-off date, whereas patient numbers were recorded on a continuous basis in the on-site clinics with a unique identifier (ID) assigned to each patient presenting at the health care centre throughout a given month. While unique ID assigned to each patient in the EHR helped to determine patient numbers accurately, the census approach of capturing occupancy data is prone to underestimation. In refugee centres with high turnover (e.g. in REG), some kind of inaccuracy through underestimating the “true” denominator cannot be ruled out, resulting for example in ratios between patients and occupancy greater than one.

The analysis is also limited by the challenges associated with the use of medical routine data. This includes issues of completeness (e.g. patient contacts may not be recorded or diagnoses of digestive diseases may be omitted) and the quality and comparability of recording practices of health care professionals as well as their objectivity and reliability [1, 26]. For a discussion of this and other limitations inherent in the presented analysis approach, see [27] and [12].

## Conclusion

Building on an empirically derived ratio between patient numbers and occupancy numbers in refugee centres, which depends on socio-demographics and centre typology, we were able to mitigate the denominator problem in refugee centres for which no data on occupancy was available. This helped to obtain estimates for the “population at risk” in order to calculate incidence rates for a selected health outcome (diseases of the digestive system). This helped to improve analysis of incidence rates over time and across centres by avoiding overestimation of disease frequency through usage of patient numbers as denominators. Additionally, predictions of disease incidence were possible based on occupancy data for centres which had no data on the health outcome of interest selected for this study. The approach could help mitigate challenges created by the denominator problem in settings with fragmented records for health and immigration data. However, the predictions could be improved in the future by obtaining and including data on pre-, peri-, and post-migration factors into the developed models.

### Electronic supplementary material

Below is the link to the electronic supplementary material.

Supplementary Material 1

## Data Availability

The datasets generated and/or analysed during the current study are not publicly available due to the data use and access regulations of the PriCare*net* Consortium. The generated and analysed datasets are available for scientific purposes from the PriCare*net* Consortium on reasonable request by contacting the spokesperson (Prof. Dr. Kayvan Bozorgmehr, refcare.allmed@med.uni-heidelberg.de).

## Abbreviations

AIC: Akaike information criterion
AR(1): First-order autocorrelation
CI: Confidence interval
ID: Unique identifier
min-max: Minimum and maximum
n_occup: Occupancy number living in a centre per month
n_pat: Number of patients in a centre per month
NSZ: Non structural zeros
Q1-Q3: Interquartile range
REC: Reception centres
ref: Reference category
REG: State-level registration
sd: Standard deviation
