# Supplementary material for: Overcoming denominator problems in refugee settings with fragmented electronic records for health and immigration data: a prediction-based approach
Source: BMC Med Res Methodol. 2024 Apr 1;24:81. doi: 10.1186/s12874-024-02204-7 (PMC10983725; doi:10.1186/s12874-024-02204-7)
Supplement: Supplementary file 1 — Supplementary Material 1 [file 12874_2024_2204_MOESM1_ESM.docx]

### **Appendix**

[A.1 More detailed description of the data used in this study 1](#_Toc158719275)

[A.2 Original R Output of the fitted models and diagnostics 11](#_Toc158719276)

[A.2.1 Negative binominal model fitted on the EHR data 11](#_Toc158719277)

[A.2.2 Generalized linear model fitted on the matched data (occup🡪pat) 13](#_Toc158719278)

[A.2.3 Generalized linear model fitted on the matched data (pat🡪occup) 13](#_Toc158719279)

[A.3 Results of sensitivity analysis 14](#_Toc158719280)

[A.3.1 Sensitivity analysis not excluding $\mathbf{noccup<npat}$ 14](#_Toc158719281)

[A.3.2 Sensitivity analysis excluding observations with 0 cases of diseases of the digestive system 18](#_Toc158719282)

[A.3.3 Sensitivity analysis including country of origin 19](#_Toc158719283)

**A.1 More detailed description of the data used in this study**

**Table A1: Description of EHR data.** Absolute and relative frequencies and mean, standard deviation, median and interquartile range (Q1 - Q3), minimum and maximum of the observed variables of the EHR data set stratified by the type of centre are given, respectively.

| Variables | REC | REG | Total |
| --- | --- | --- | --- |
|  | (N=345) | (N=100) | (N=445) |
| **Centre** |  |  |  |
| 1 | 22 (6%) | 0 (0%) | 22 (5%) |
| 2 | 0 (0%) | 6 (6%) | 6 (1%) |
| 4 | 37 (11%) | 0 (0%) | 37 (8%) |
| 5 | 22 (6%) | 0 (0%) | 22 (5%) |
| 6 | 32 (9%) | 0 (0%) | 32 (7%) |
| 7 | 0 (0%) | 38 (38%) | 38 (9%) |
| 8 | 7 (2%) | 0 (0%) | 7 (2%) |
| 9 | 0 (0%) | 23 (23%) | 23 (5%) |
| 10 | 19 (6%) | 0 (0%) | 19 (4%) |
| 11 | 27 (8%) | 0 (0%) | 27 (6%) |
| 12 | 16 (5%) | 0 (0%) | 16 (4%) |
| 13 | 0 (0%) | 27 (27%) | 27 (6%) |
| 14 | 16 (5%) | 0 (0%) | 16 (4%) |
| 15 | 17 (5%) | 0 (0%) | 17 (4%) |
| 17 | 25 (7%) | 0 (0%) | 25 (6%) |
| 18 | 16 (5%) | 0 (0%) | 16 (4%) |
| 19 | 22 (6%) | 0 (0%) | 22 (5%) |
| 20 | 44 (13%) | 0 (0%) | 44 (10%) |
| 21 | 0 (0%) | 6 (6%) | 6 (1%) |
| 22 | 5 (1%) | 0 (0%) | 5 (1%) |
| 23 | 18 (5%) | 0 (0%) | 18 (4%) |
| **Month** |  |  |  |
| 2017 11 | 1 (0%) | 0 (0%) | 1 (0%) |
| 2017 12 | 1 (0%) | 0 (0%) | 1 (0%) |
| 2018 01 | 1 (0%) | 0 (0%) | 1 (0%) |
| 2018 02 | 1 (0%) | 0 (0%) | 1 (0%) |
| 2018 03 | 1 (0%) | 0 (0%) | 1 (0%) |
| 2018 04 | 3 (1%) | 0 (0%) | 3 (1%) |
| 2018 05 | 3 (1%) | 1 (1%) | 4 (1%) |
| 2018 06 | 4 (1%) | 1 (1%) | 5 (1%) |
| 2018 07 | 5 (1%) | 1 (1%) | 6 (1%) |
| 2018 08 | 6 (2%) | 2 (2%) | 8 (2%) |
| 2018 09 | 6 (2%) | 2 (2%) | 8 (2%) |
| 2018 10 | 6 (2%) | 2 (2%) | 8 (2%) |
| 2018 11 | 6 (2%) | 2 (2%) | 8 (2%) |
| 2018 12 | 7 (2%) | 2 (2%) | 9 (2%) |
| 2019 01 | 7 (2%) | 2 (2%) | 9 (2%) |
| 2019 02 | 7 (2%) | 2 (2%) | 9 (2%) |
| 2019 03 | 7 (2%) | 2 (2%) | 9 (2%) |
| 2019 04 | 7 (2%) | 3 (3%) | 10 (2%) |
| 2019 05 | 7 (2%) | 3 (3%) | 10 (2%) |
| 2019 06 | 8 (2%) | 3 (3%) | 11 (2%) |
| 2019 07 | 9 (3%) | 3 (3%) | 12 (3%) |
| 2019 08 | 8 (2%) | 3 (3%) | 11 (2%) |
| 2019 09 | 11 (3%) | 3 (3%) | 14 (3%) |
| 2019 10 | 12 (3%) | 3 (3%) | 15 (3%) |
| 2019 11 | 11 (3%) | 3 (3%) | 14 (3%) |
| 2019 12 | 11 (3%) | 3 (3%) | 14 (3%) |
| 2020 01 | 11 (3%) | 3 (3%) | 14 (3%) |
| 2020 02 | 11 (3%) | 3 (3%) | 14 (3%) |
| 2020 03 | 12 (3%) | 3 (3%) | 15 (3%) |
| 2020 04 | 10 (3%) | 3 (3%) | 13 (3%) |
| 2020 05 | 11 (3%) | 3 (3%) | 14 (3%) |
| 2020 06 | 11 (3%) | 3 (3%) | 14 (3%) |
| 2020 07 | 10 (3%) | 2 (2%) | 12 (3%) |
| 2020 08 | 9 (3%) | 2 (2%) | 11 (2%) |
| 2020 09 | 9 (3%) | 2 (2%) | 11 (2%) |
| 2020 10 | 9 (3%) | 2 (2%) | 11 (2%) |
| 2020 11 | 9 (3%) | 2 (2%) | 11 (2%) |
| 2020 12 | 10 (3%) | 2 (2%) | 12 (3%) |
| 2021 01 | 10 (3%) | 4 (4%) | 14 (3%) |
| 2021 02 | 11 (3%) | 4 (4%) | 15 (3%) |
| 2021 03 | 11 (3%) | 4 (4%) | 15 (3%) |
| 2021 04 | 11 (3%) | 4 (4%) | 15 (3%) |
| 2021 05 | 11 (3%) | 4 (4%) | 15 (3%) |
| 2021 06 | 11 (3%) | 4 (4%) | 15 (3%) |
| 2021 07 | 2 (1%) | 0 (0%) | 2 (0%) |
| **Male (patients)** |  |  |  |
| N | 345 | 100 | 445 |
| mean | 60 | 60 | 60 |
| sd | 15 | 7.4 | 14 |
| median | 61 | 57 | 60 |
| Q1 - Q3 | 54 -- 69 | 54 -- 64 | 54 -- 68 |
| min - max | 0 -- 100 | 47 -- 83 | 0 -- 100 |
| **Adult (patients)** |  |  |  |
| N | 345 | 100 | 445 |
| mean | 79 | 83 | 80 |
| sd | 11 | 5.7 | 10 |
| median | 81 | 82 | 81 |
| Q1 - Q3 | 73 -- 86 | 78 -- 87 | 74 -- 87 |
| min - max | 0 -- 100 | 70 -- 94 | 0 -- 100 |
| **Diseases of the digestive system** |  |  |  |
| N | 345 | 100 | 445 |
| mean | 17 | 41 | 23 |
| sd | 21 | 23 | 23 |
| median | 8 | 36 | 12 |
| Q1 - Q3 | 4 -- 22 | 22 -- 60 | 5 -- 35 |
| min - max | 0 -- 85 | 3 -- 89 | 0 -- 89 |
| **n_pat** |  |  |  |
| N | 345 | 100 | 445 |
| mean | 182 | 396 | 230 |
| sd | 172 | 209 | 202 |
| median | 117 | 360 | 149 |
| Q1 - Q3 | 80 -- 208 | 212 -- 588 | 93 -- 304 |
| min - max | 5 -- 934 | 29 -- 934 | 5 -- 934 |
| **Incidence of diseases of the digestive system with respect to patients** |  |  |  |
| N | 345 | 100 | 445 |
| mean | 0.089 | 0.1 | 0.092 |
| sd | 0.065 | 0.028 | 0.059 |
| median | 0.084 | 0.1 | 0.092 |
| Q1 - Q3 | 0.042 -- 0.13 | 0.088 -- 0.12 | 0.056 -- 0.12 |
| min - max | 0 -- 0.3 | 0.032 -- 0.21 | 0 -- 0.3 |

**Table A2: Description of occupancy data.** Absolute and relative frequencies and mean, standard deviation, median and interquartile range (Q1 - Q3), minimum and maximum of the observed variables of the occupancy data set stratified by the type of centre are given, respectively.

| Variables | REC | REG | Total |
| --- | --- | --- | --- |
|  | (N=137) | (N=78) | (N=215) |
| **Centre** |  |  |  |
| 1 | 5 (4%) | 0 (0%) | 5 (2%) |
| 2 | 0 (0%) | 8 (10%) | 8 (4%) |
| 3 | 1 (1%) | 0 (0%) | 1 (0%) |
| 4 | 17 (12%) | 0 (0%) | 17 (8%) |
| 5 | 13 (9%) | 0 (0%) | 13 (6%) |
| 6 | 19 (14%) | 0 (0%) | 19 (9%) |
| 7 | 0 (0%) | 33 (42%) | 33 (15%) |
| 8 | 1 (1%) | 0 (0%) | 1 (0%) |
| 9 | 0 (0%) | 14 (18%) | 14 (7%) |
| 10 | 14 (10%) | 0 (0%) | 14 (7%) |
| 11 | 14 (10%) | 0 (0%) | 14 (7%) |
| 13 | 0 (0%) | 14 (18%) | 14 (7%) |
| 14 | 17 (12%) | 0 (0%) | 17 (8%) |
| 15 | 12 (9%) | 0 (0%) | 12 (6%) |
| 16 | 0 (0%) | 1 (1%) | 1 (0%) |
| 17 | 18 (13%) | 0 (0%) | 18 (8%) |
| 18 | 4 (3%) | 0 (0%) | 4 (2%) |
| 19 | 2 (1%) | 0 (0%) | 2 (1%) |
| 21 | 0 (0%) | 8 (10%) | 8 (4%) |
| **Month** |  |  |  |
| 2018 10 | 2 (1%) | 1 (1%) | 3 (1%) |
| 2018 11 | 1 (1%) | 0 (0%) | 1 (0%) |
| 2018 12 | 4 (3%) | 2 (3%) | 6 (3%) |
| 2019 01 | 5 (4%) | 2 (3%) | 7 (3%) |
| 2019 02 | 3 (2%) | 1 (1%) | 4 (2%) |
| 2019 03 | 6 (4%) | 2 (3%) | 8 (4%) |
| 2019 04 | 2 (1%) | 0 (0%) | 2 (1%) |
| 2019 05 | 4 (3%) | 1 (1%) | 5 (2%) |
| 2019 06 | 5 (4%) | 2 (3%) | 7 (3%) |
| 2019 07 | 4 (3%) | 1 (1%) | 5 (2%) |
| 2019 08 | 5 (4%) | 2 (3%) | 7 (3%) |
| 2019 09 | 5 (4%) | 2 (3%) | 7 (3%) |
| 2019 10 | 7 (5%) | 2 (3%) | 9 (4%) |
| 2019 11 | 7 (5%) | 1 (1%) | 8 (4%) |
| 2019 12 | 7 (5%) | 2 (3%) | 9 (4%) |
| 2020 01 | 7 (5%) | 2 (3%) | 9 (4%) |
| 2020 02 | 7 (5%) | 3 (4%) | 10 (5%) |
| 2020 03 | 3 (2%) | 1 (1%) | 4 (2%) |
| 2020 04 | 5 (4%) | 3 (4%) | 8 (4%) |
| 2020 05 | 5 (4%) | 3 (4%) | 8 (4%) |
| 2020 06 | 5 (4%) | 3 (4%) | 8 (4%) |
| 2020 07 | 6 (4%) | 2 (3%) | 8 (4%) |
| 2020 08 | 0 (0%) | 1 (1%) | 1 (0%) |
| 2020 09 | 3 (2%) | 1 (1%) | 4 (2%) |
| 2020 10 | 1 (1%) | 1 (1%) | 2 (1%) |
| 2020 11 | 4 (3%) | 2 (3%) | 6 (3%) |
| 2020 12 | 0 (0%) | 1 (1%) | 1 (0%) |
| 2021 01 | 3 (2%) | 2 (3%) | 5 (2%) |
| 2021 02 | 0 (0%) | 3 (4%) | 3 (1%) |
| 2021 03 | 4 (3%) | 4 (5%) | 8 (4%) |
| 2021 04 | 2 (1%) | 4 (5%) | 6 (3%) |
| 2021 05 | 3 (2%) | 4 (5%) | 7 (3%) |
| 2021 06 | 2 (1%) | 4 (5%) | 6 (3%) |
| 2021 07 | 3 (2%) | 4 (5%) | 7 (3%) |
| 2021 08 | 4 (3%) | 4 (5%) | 8 (4%) |
| 2021 09 | 3 (2%) | 4 (5%) | 7 (3%) |
| 2021 10 | 0 (0%) | 1 (1%) | 1 (0%) |
| **Male (occupancy)** |  |  |  |
| N | 137 | 78 | 215 |
| mean | 67 | 65 | 66 |
| sd | 16 | 7 | 14 |
| median | 67 | 63 | 65 |
| Q1 - Q3 | 53 -- 78 | 61 -- 70 | 59 -- 74 |
| min - max | 24 -- 98 | 49 -- 97 | 24 -- 98 |
| **Adult (occupancy)** |  |  |  |
| N | 137 | 78 | 215 |
| mean | 79 | 75 | 77 |
| sd | 11 | 8.5 | 10 |
| median | 79 | 74 | 77 |
| Q1 - Q3 | 69 -- 87 | 70 -- 79 | 70 -- 84 |
| min - max | 54 -- 100 | 55 -- 114 | 54 -- 114 |
| **n_occup** |  |  |  |
| N | 137 | 78 | 215 |
| mean | 253 | 515 | 348 |
| sd | 138 | 389 | 287 |
| median | 226 | 362 | 244 |
| Q1 - Q3 | 161 -- 289 | 190 -- 847 | 164 -- 449 |
| min - max | 32 -- 600 | 72 -- 1516 | 32 -- 1516 |

**Table A3: Description of matched data.** Absolute and relative frequencies and mean, standard deviation, median and interquartile range (Q1 - Q3), minimum and maximum of the observed variables of the matched data set stratified by the type of centre are given, respectively.

| Variables | REC | REG | Total |
| --- | --- | --- | --- |
|  | (N=104) | (N=43) | (N=147) |
| **Month** |  |  |  |
| 2017 11 | 0 (0%) | 0 (0%) | 0 (0%) |
| 2017 12 | 0 (0%) | 0 (0%) | 0 (0%) |
| 2018 01 | 0 (0%) | 0 (0%) | 0 (0%) |
| 2018 02 | 0 (0%) | 0 (0%) | 0 (0%) |
| 2018 03 | 0 (0%) | 0 (0%) | 0 (0%) |
| 2018 04 | 0 (0%) | 0 (0%) | 0 (0%) |
| 2018 05 | 0 (0%) | 0 (0%) | 0 (0%) |
| 2018 06 | 0 (0%) | 0 (0%) | 0 (0%) |
| 2018 07 | 0 (0%) | 0 (0%) | 0 (0%) |
| 2018 08 | 0 (0%) | 0 (0%) | 0 (0%) |
| 2018 09 | 0 (0%) | 0 (0%) | 0 (0%) |
| 2018 10 | 2 (2%) | 1 (2%) | 3 (2%) |
| 2018 11 | 1 (1%) | 0 (0%) | 1 (1%) |
| 2018 12 | 4 (4%) | 1 (2%) | 5 (3%) |
| 2019 01 | 5 (5%) | 1 (2%) | 6 (4%) |
| 2019 02 | 3 (3%) | 0 (0%) | 3 (2%) |
| 2019 03 | 5 (5%) | 1 (2%) | 6 (4%) |
| 2019 04 | 2 (2%) | 0 (0%) | 2 (1%) |
| 2019 05 | 4 (4%) | 1 (2%) | 5 (3%) |
| 2019 06 | 5 (5%) | 2 (5%) | 7 (5%) |
| 2019 07 | 4 (4%) | 1 (2%) | 5 (3%) |
| 2019 08 | 5 (5%) | 1 (2%) | 6 (4%) |
| 2019 09 | 5 (5%) | 2 (5%) | 7 (5%) |
| 2019 10 | 5 (5%) | 1 (2%) | 6 (4%) |
| 2019 11 | 6 (6%) | 1 (2%) | 7 (5%) |
| 2019 12 | 5 (5%) | 2 (5%) | 7 (5%) |
| 2020 01 | 4 (4%) | 1 (2%) | 5 (3%) |
| 2020 02 | 5 (5%) | 1 (2%) | 6 (4%) |
| 2020 03 | 2 (2%) | 1 (2%) | 3 (2%) |
| 2020 04 | 3 (3%) | 2 (5%) | 5 (3%) |
| 2020 05 | 3 (3%) | 3 (7%) | 6 (4%) |
| 2020 06 | 4 (4%) | 3 (7%) | 7 (5%) |
| 2020 07 | 3 (3%) | 1 (2%) | 4 (3%) |
| 2020 08 | 0 (0%) | 1 (2%) | 1 (1%) |
| 2020 09 | 3 (3%) | 0 (0%) | 3 (2%) |
| 2020 10 | 1 (1%) | 0 (0%) | 1 (1%) |
| 2020 11 | 3 (3%) | 2 (5%) | 5 (3%) |
| 2020 12 | 0 (0%) | 1 (2%) | 1 (1%) |
| 2021 01 | 2 (2%) | 2 (5%) | 4 (3%) |
| 2021 02 | 0 (0%) | 2 (5%) | 2 (1%) |
| 2021 03 | 3 (3%) | 2 (5%) | 5 (3%) |
| 2021 04 | 2 (2%) | 2 (5%) | 4 (3%) |
| 2021 05 | 2 (2%) | 2 (5%) | 4 (3%) |
| 2021 06 | 2 (2%) | 2 (5%) | 4 (3%) |
| 2021 07 | 1 (1%) | 0 (0%) | 1 (1%) |
| **Centre** |  |  |  |
| 1 | 3 (3%) | 0 (0%) | 3 (2%) |
| 2 | 0 (0%) | 2 (5%) | 2 (1%) |
| 4 | 11 (11%) | 0 (0%) | 11 (7%) |
| 5 | 13 (12%) | 0 (0%) | 13 (9%) |
| 6 | 15 (14%) | 0 (0%) | 15 (10%) |
| 7 | 0 (0%) | 27 (63%) | 27 (18%) |
| 8 | 1 (1%) | 0 (0%) | 1 (1%) |
| 9 | 0 (0%) | 5 (12%) | 5 (3%) |
| 10 | 7 (7%) | 0 (0%) | 7 (5%) |
| 11 | 13 (12%) | 0 (0%) | 13 (9%) |
| 12 | 0 (0%) | 0 (0%) | 0 (0%) |
| 13 | 0 (0%) | 9 (21%) | 9 (6%) |
| 14 | 9 (9%) | 0 (0%) | 9 (6%) |
| 15 | 11 (11%) | 0 (0%) | 11 (7%) |
| 17 | 16 (15%) | 0 (0%) | 16 (11%) |
| 18 | 4 (4%) | 0 (0%) | 4 (3%) |
| 19 | 1 (1%) | 0 (0%) | 1 (1%) |
| 20 | 0 (0%) | 0 (0%) | 0 (0%) |
| 21 | 0 (0%) | 0 (0%) | 0 (0%) |
| 22 | 0 (0%) | 0 (0%) | 0 (0%) |
| 23 | 0 (0%) | 0 (0%) | 0 (0%) |
| **Male (patients)** |  |  |  |
| N | 104 | 43 | 147 |
| mean | 60 | 60 | 60 |
| sd | 14 | 7.4 | 13 |
| median | 59 | 57 | 58 |
| Q1 - Q3 | 49 -- 70 | 55 -- 65 | 52 -- 69 |
| min - max | 23 -- 90 | 47 -- 79 | 23 -- 90 |
| **Adult (patients)** |  |  |  |
| N | 104 | 43 | 147 |
| mean | 77 | 85 | 80 |
| sd | 11 | 4.8 | 10 |
| median | 77 | 85 | 81 |
| Q1 - Q3 | 70 -- 86 | 82 -- 88 | 72 -- 87 |
| min - max | 48 -- 100 | 71 -- 94 | 48 -- 100 |
| **Diseases of the digestive system** |  |  |  |
| N | 104 | 43 | 147 |
| mean | 10 | 47 | 21 |
| sd | 10 | 24 | 23 |
| median | 7 | 45 | 11 |
| Q1 - Q3 | 4 -- 13 | 24 -- 68 | 5 -- 31 |
| min - max | 0 -- 45 | 5 -- 83 | 0 -- 83 |
| **n_pat** |  |  |  |
| N | 104 | 43 | 147 |
| mean | 118 | 441 | 212 |
| sd | 55 | 223 | 195 |
| median | 105 | 463 | 127 |
| Q1 - Q3 | 80 -- 140 | 204 -- 636 | 95 -- 227 |
| min - max | 29 -- 274 | 101 -- 934 | 29 -- 934 |
| **Incidence of diseases of the digestive system with respect to patients** |  |  |  |
| N | 104 | 43 | 147 |
| mean | 0.08 | 0.11 | 0.088 |
| sd | 0.057 | 0.023 | 0.051 |
| median | 0.075 | 0.11 | 0.094 |
| Q1 - Q3 | 0.037 -- 0.12 | 0.094 -- 0.12 | 0.054 -- 0.12 |
| min - max | 0 -- 0.23 | 0.05 -- 0.16 | 0 -- 0.23 |
| **Male (occupation)** |  |  |  |
| N | 104 | 43 | 147 |
| mean | 65 | 66 | 65 |
| sd | 15 | 8.3 | 13 |
| median | 66 | 64 | 65 |
| Q1 - Q3 | 50 -- 76 | 60 -- 72 | 57 -- 75 |
| min - max | 35 -- 94 | 49 -- 97 | 35 -- 97 |
| **Adult (occupation)** |  |  |  |
| N | 104 | 43 | 147 |
| mean | 78 | 76 | 77 |
| sd | 10 | 9 | 10 |
| median | 78 | 75 | 77 |
| Q1 - Q3 | 69 -- 85 | 71 -- 79 | 69 -- 84 |
| min - max | 54 -- 100 | 61 -- 114 | 54 -- 114 |
| **n_occup** |  |  |  |
| N | 104 | 43 | 147 |
| mean | 270 | 695 | 394 |
| sd | 142 | 391 | 310 |
| median | 231 | 769 | 276 |
| Q1 - Q3 | 168 -- 298 | 352 -- 1026 | 186 -- 503 |
| min - max | 32 -- 600 | 128 -- 1516 | 32 -- 1516 |
| **Incidence of diseases of the digestive system with respect to occupation** |  |  |  |
| N | 104 | 43 | 147 |
| mean | 0.036 | 0.071 | 0.047 |
| sd | 0.031 | 0.019 | 0.032 |
| median | 0.031 | 0.069 | 0.043 |
| Q1 - Q3 | 0.017 -- 0.049 | 0.055 -- 0.081 | 0.024 -- 0.068 |
| min - max | 0 -- 0.14 | 0.037 -- 0.12 | 0 -- 0.14 |
| **ratio** |  |  |  |
| N | 104 | 43 | 147 |
| mean | 0.49 | 0.68 | 0.54 |
| sd | 0.18 | 0.16 | 0.2 |
| median | 0.43 | 0.67 | 0.49 |
| Q1 - Q3 | 0.37 -- 0.57 | 0.53 -- 0.78 | 0.39 -- 0.69 |
| min - max | 0.13 -- 0.99 | 0.37 -- 0.99 | 0.13 -- 0.99 |

**Table A4: Description of unmatched EHR data.** Absolute and relative frequencies and mean, standard deviation, median and interquartile range (Q1 - Q3), minimum and maximum of the observed/predicted variables of the unmatched EHR data set are given, respectively. In case the predicted variables are described, boxes indicate the model and data set used ($\cdot\cdot\cdot\cdot\cdot$: predictions made on the basis of model of Section 2.3.3 modelling relationship 3 (patient-occupancy ratio when patient data is given)).

| Variables | REC | REG | Total |
| --- | --- | --- | --- |
|  | (N=234) | (N=36) | (N=270) |
| **Centre** |  |  |  |
| 1 | 19 (8%) | 0 (0%) | 19 (7%) |
| 2 | 0 (0%) | 1 (3%) | 1 (0%) |
| 4 | 21 (9%) | 0 (0%) | 21 (8%) |
| 5 | 9 (4%) | 0 (0%) | 9 (3%) |
| 6 | 15 (6%) | 0 (0%) | 15 (6%) |
| 7 | 0 (0%) | 8 (22%) | 8 (3%) |
| 8 | 6 (3%) | 0 (0%) | 6 (2%) |
| 9 | 0 (0%) | 10 (28%) | 10 (4%) |
| 10 | 12 (5%) | 0 (0%) | 12 (4%) |
| 11 | 14 (6%) | 0 (0%) | 14 (5%) |
| 12 | 16 (7%) | 0 (0%) | 16 (6%) |
| 13 | 0 (0%) | 16 (44%) | 16 (6%) |
| 14 | 7 (3%) | 0 (0%) | 7 (3%) |
| 15 | 6 (3%) | 0 (0%) | 6 (2%) |
| 17 | 9 (4%) | 0 (0%) | 9 (3%) |
| 18 | 12 (5%) | 0 (0%) | 12 (4%) |
| 19 | 21 (9%) | 0 (0%) | 21 (8%) |
| 20 | 44 (19%) | 0 (0%) | 44 (16%) |
| 21 | 0 (0%) | 1 (3%) | 1 (0%) |
| 22 | 5 (2%) | 0 (0%) | 5 (2%) |
| 23 | 18 (8%) | 0 (0%) | 18 (7%) |
| **Month** |  |  |  |
| 2017 11 | 1 (0%) | 0 (0%) | 1 (0%) |
| 2017 12 | 1 (0%) | 0 (0%) | 1 (0%) |
| 2018 01 | 1 (0%) | 0 (0%) | 1 (0%) |
| 2018 02 | 1 (0%) | 0 (0%) | 1 (0%) |
| 2018 03 | 1 (0%) | 0 (0%) | 1 (0%) |
| 2018 04 | 3 (1%) | 0 (0%) | 3 (1%) |
| 2018 05 | 3 (1%) | 1 (3%) | 4 (1%) |
| 2018 06 | 4 (2%) | 1 (3%) | 5 (2%) |
| 2018 07 | 5 (2%) | 1 (3%) | 6 (2%) |
| 2018 08 | 6 (3%) | 2 (6%) | 8 (3%) |
| 2018 09 | 6 (3%) | 2 (6%) | 8 (3%) |
| 2018 10 | 4 (2%) | 1 (3%) | 5 (2%) |
| 2018 11 | 5 (2%) | 2 (6%) | 7 (3%) |
| 2018 12 | 3 (1%) | 0 (0%) | 3 (1%) |
| 2019 01 | 2 (1%) | 0 (0%) | 2 (1%) |
| 2019 02 | 4 (2%) | 1 (3%) | 5 (2%) |
| 2019 03 | 1 (0%) | 0 (0%) | 1 (0%) |
| 2019 04 | 5 (2%) | 3 (8%) | 8 (3%) |
| 2019 05 | 3 (1%) | 2 (6%) | 5 (2%) |
| 2019 06 | 3 (1%) | 1 (3%) | 4 (1%) |
| 2019 07 | 5 (2%) | 2 (6%) | 7 (3%) |
| 2019 08 | 3 (1%) | 1 (3%) | 4 (1%) |
| 2019 09 | 6 (3%) | 1 (3%) | 7 (3%) |
| 2019 10 | 6 (3%) | 1 (3%) | 7 (3%) |
| 2019 11 | 5 (2%) | 2 (6%) | 7 (3%) |
| 2019 12 | 6 (3%) | 1 (3%) | 7 (3%) |
| 2020 01 | 7 (3%) | 1 (3%) | 8 (3%) |
| 2020 02 | 6 (3%) | 0 (0%) | 6 (2%) |
| 2020 03 | 10 (4%) | 2 (6%) | 12 (4%) |
| 2020 04 | 7 (3%) | 0 (0%) | 7 (3%) |
| 2020 05 | 8 (3%) | 0 (0%) | 8 (3%) |
| 2020 06 | 7 (3%) | 0 (0%) | 7 (3%) |
| 2020 07 | 6 (3%) | 1 (3%) | 7 (3%) |
| 2020 08 | 9 (4%) | 1 (3%) | 10 (4%) |
| 2020 09 | 6 (3%) | 1 (3%) | 7 (3%) |
| 2020 10 | 8 (3%) | 1 (3%) | 9 (3%) |
| 2020 11 | 5 (2%) | 0 (0%) | 5 (2%) |
| 2020 12 | 10 (4%) | 1 (3%) | 11 (4%) |
| 2021 01 | 7 (3%) | 2 (6%) | 9 (3%) |
| 2021 02 | 11 (5%) | 1 (3%) | 12 (4%) |
| 2021 03 | 7 (3%) | 0 (0%) | 7 (3%) |
| 2021 04 | 9 (4%) | 0 (0%) | 9 (3%) |
| 2021 05 | 8 (3%) | 0 (0%) | 8 (3%) |
| 2021 06 | 9 (4%) | 0 (0%) | 9 (3%) |
| 2021 07 | 1 (0%) | 0 (0%) | 1 (0%) |
| **Male (patients)** |  |  |  |
| N | 234 | 36 | 270 |
| mean | 60 | 58 | 59 |
| sd | 15 | 6.5 | 14 |
| median | 60 | 56 | 60 |
| Q1 - Q3 | 54 -- 68 | 53 -- 61 | 54 -- 67 |
| min - max | 0 -- 100 | 51 -- 83 | 0 -- 100 |
| **Adults (patients)** |  |  |  |
| N | 234 | 36 | 270 |
| mean | 80 | 82 | 80 |
| sd | 11 | 5.9 | 11 |
| median | 81 | 82 | 81 |
| Q1 - Q3 | 74 -- 86 | 77 -- 87 | 75 -- 86 |
| min - max | 0 -- 100 | 70 -- 93 | 0 -- 100 |
| **Diseases of the digestive system** |  |  |  |
| N | 234 | 36 | 270 |
| mean | 21 | 37 | 23 |
| sd | 23 | 23 | 24 |
| median | 11 | 30 | 12 |
| Q1 - Q3 | 4 -- 34 | 21 -- 44 | 5 -- 37 |
| min - max | 0 -- 85 | 3 -- 89 | 0 -- 89 |
| **n_pat** |  |  |  |
| N | 234 | 36 | 270 |
| mean | 211 | 373 | 233 |
| sd | 199 | 210 | 208 |
| median | 132 | 306 | 150 |
| Q1 - Q3 | 80 -- 275 | 224 -- 470 | 85 -- 316 |
| min - max | 5 -- 934 | 29 -- 793 | 5 -- 934 |
| **Incidence of diseases of the digestive system with respect to patients** |  |  |  |
| N | 234 | 36 | 270 |
| mean | 0.095 | 0.1 | 0.096 |
| sd | 0.068 | 0.032 | 0.064 |
| median | 0.09 | 0.1 | 0.092 |
| Q1 - Q3 | 0.052 -- 0.13 | 0.083 -- 0.12 | 0.057 -- 0.13 |
| min - max | 0 -- 0.3 | 0.032 -- 0.21 | 0 -- 0.3 |
| **ratio** |  |  |  |
| N | 234 | 36 | 270 |
| mean | 0.48 | 0.67 | 0.51 |
| sd | 0.054 | 0.025 | 0.083 |
| median | 0.49 | 0.67 | 0.5 |
| Q1 - Q3 | 0.46 -- 0.51 | 0.65 -- 0.68 | 0.47 -- 0.53 |
| min - max | 0.23 -- 0.63 | 0.64 -- 0.77 | 0.23 -- 0.77 |
| **n_occup** |  |  |  |
| N | 234 | 36 | 270 |
| mean | 431 | 566 | 454 |
| sd | 400 | 336 | 404 |
| median | 266 | 459 | 287 |
| Q1 - Q3 | 165 -- 535 | 334 -- 714 | 182 -- 585 |
| min - max | 14 -- 1821 | 37 -- 1246 | 12 -- 1876 |
| **Incidence of diseases of the digestive system with respect to occupation** |  |  |  |
| N | 234 | 36 | 270 |
| mean | 0.045 | 0.069 | 0.048 |
| sd | 0.033 | 0.023 | 0.033 |
| median | 0.042 | 0.068 | 0.045 |
| Q1 - Q3 | 0.023 -- 0.062 | 0.056 -- 0.078 | 0.027 -- 0.068 |
| min - max | 0 -- 0.16 | 0.021 -- 0.16 | 0 -- 0.16 |

**Table A5: Description of unmatched occupancy data.** Absolute and relative frequencies and mean, standard deviation, median and interquartile range (Q1 - Q3), minimum and maximum of the observed/predicted variables of the unmatched occupancy data sets are given, respectively. In case the predicted variables are described, boxes indicate the model and data set used (­– - – - –: predictions made on the basis of model of Section 2.3.2 and 2.3.1 modelling relationships 2 (patient-occupancy ratio when occupancy data is given) and 1 (disease incidence); - - - - -: predictions made on the basis of model of Section 2.3.2 modelling relationship 2 (patient-occupancy ratio when occupancy data is given)).

| Variables | REC | REG | Total |
| --- | --- | --- | --- |
|  | (N=26) | (N=14) | (N=40) |
| **Centre** |  |  |  |
| 1 | 2 (8%) | 0 (0%) | 2 (5%) |
| 2 | 0 (0%) | 3 (21%) | 3 (8%) |
| 3 | 1 (4%) | 0 (0%) | 1 (2%) |
| 4 | 1 (4%) | 0 (0%) | 1 (2%) |
| 5 | 0 (0%) | 0 (0%) | 0 (0%) |
| 6 | 2 (8%) | 0 (0%) | 2 (5%) |
| 7 | 0 (0%) | 3 (21%) | 3 (8%) |
| 8 | 0 (0%) | 0 (0%) | 0 (0%) |
| 9 | 0 (0%) | 1 (7%) | 1 (2%) |
| 10 | 7 (27%) | 0 (0%) | 7 (18%) |
| 11 | 1 (4%) | 0 (0%) | 1 (2%) |
| 13 | 0 (0%) | 3 (21%) | 3 (8%) |
| 14 | 8 (31%) | 0 (0%) | 8 (20%) |
| 15 | 1 (4%) | 0 (0%) | 1 (2%) |
| 16 | 0 (0%) | 1 (7%) | 1 (2%) |
| 17 | 2 (8%) | 0 (0%) | 2 (5%) |
| **Month** |  |  |  |
| 2018 10 | 0 (0%) | 0 (0%) | 0 (0%) |
| 2018 11 | 0 (0%) | 0 (0%) | 0 (0%) |
| 2018 12 | 0 (0%) | 0 (0%) | 0 (0%) |
| 2019 01 | 0 (0%) | 0 (0%) | 0 (0%) |
| 2019 02 | 0 (0%) | 0 (0%) | 0 (0%) |
| 2019 03 | 0 (0%) | 0 (0%) | 0 (0%) |
| 2019 04 | 0 (0%) | 0 (0%) | 0 (0%) |
| 2019 05 | 0 (0%) | 0 (0%) | 0 (0%) |
| 2019 06 | 0 (0%) | 0 (0%) | 0 (0%) |
| 2019 07 | 0 (0%) | 0 (0%) | 0 (0%) |
| 2019 08 | 0 (0%) | 0 (0%) | 0 (0%) |
| 2019 09 | 0 (0%) | 0 (0%) | 0 (0%) |
| 2019 10 | 1 (4%) | 0 (0%) | 1 (2%) |
| 2019 11 | 1 (4%) | 0 (0%) | 1 (2%) |
| 2019 12 | 2 (8%) | 0 (0%) | 2 (5%) |
| 2020 01 | 3 (12%) | 0 (0%) | 3 (8%) |
| 2020 02 | 2 (8%) | 0 (0%) | 2 (5%) |
| 2020 03 | 1 (4%) | 0 (0%) | 1 (2%) |
| 2020 04 | 2 (8%) | 0 (0%) | 2 (5%) |
| 2020 05 | 2 (8%) | 0 (0%) | 2 (5%) |
| 2020 06 | 1 (4%) | 0 (0%) | 1 (2%) |
| 2020 07 | 2 (8%) | 1 (7%) | 3 (8%) |
| 2020 08 | 0 (0%) | 0 (0%) | 0 (0%) |
| 2020 09 | 0 (0%) | 0 (0%) | 0 (0%) |
| 2020 10 | 0 (0%) | 0 (0%) | 0 (0%) |
| 2020 11 | 0 (0%) | 0 (0%) | 0 (0%) |
| 2020 12 | 0 (0%) | 0 (0%) | 0 (0%) |
| 2021 01 | 0 (0%) | 0 (0%) | 0 (0%) |
| 2021 02 | 0 (0%) | 0 (0%) | 0 (0%) |
| 2021 03 | 0 (0%) | 0 (0%) | 0 (0%) |
| 2021 04 | 0 (0%) | 0 (0%) | 0 (0%) |
| 2021 05 | 0 (0%) | 0 (0%) | 0 (0%) |
| 2021 06 | 0 (0%) | 0 (0%) | 0 (0%) |
| 2021 07 | 2 (8%) | 4 (29%) | 6 (15%) |
| 2021 08 | 4 (15%) | 4 (29%) | 8 (20%) |
| 2021 09 | 3 (12%) | 4 (29%) | 7 (18%) |
| 2021 10 | 0 (0%) | 1 (7%) | 1 (2%) |
| **Male (occupancy)** |  |  |  |
| N | 26 | 14 | 40 |
| mean | 71 | 64 | 68 |
| sd | 21 | 4.4 | 17 |
| median | 69 | 63 | 65 |
| Q1 - Q3 | 61 -- 95 | 62 -- 66 | 61 -- 72 |
| min - max | 24 -- 98 | 57 -- 72 | 24 -- 98 |
| **Adult (occupancy)** |  |  |  |
| N | 26 | 14 | 40 |
| mean | 81 | 71 | 78 |
| sd | 13 | 7.4 | 13 |
| median | 78 | 71 | 75 |
| Q1 - Q3 | 72 -- 97 | 67 -- 74 | 69 -- 85 |
| min - max | 59 -- 100 | 58 -- 86 | 58 -- 100 |
| **n_occup** |  |  |  |
| N | 26 | 14 | 40 |
| mean | 225 | 331 | 262 |
| sd | 118 | 311 | 209 |
| median | 220 | 197 | 210 |
| Q1 - Q3 | 154 -- 277 | 125 -- 311 | 130 -- 296 |
| min - max | 56 -- 583 | 87 -- 958 | 56 -- 958 |
| **ratio** |  |  |  |
| N | 26 | 14 | 40 |
| mean | 0.52 | 0.74 | 0.6 |
| sd | 0.084 | 0.11 | 0.14 |
| median | 0.51 | 0.76 | 0.59 |
| Q1 - Q3 | 0.46 -- 0.61 | 0.65 -- 0.81 | 0.49 -- 0.67 |
| min - max | 0.38 -- 0.65 | 0.58 -- 0.91 | 0.38 -- 0.91 |
| **n_pat** |  |  |  |
| N | 26 | 14 | 40 |
| mean | 118 | 217 | 153 |
| sd | 66 | 168 | 121 |
| median | 115 | 157 | 118 |
| Q1 - Q3 | 96 -- 132 | 94 -- 223 | 95 -- 169 |
| min - max | 25 -- 343 | 73 -- 552 | 25 -- 552 |
| **Diseases of the digestive system** |  |  |  |
| N | 19 | 5 | 24 |
| Nmiss | 7 (27%) | 9 (64%) | 16 (40%) |
| mean | 16 | 20 | 17 |
| sd | 5.7 | 12 | 7.2 |
| median | 15 | 15 | 15 |
| Q1 - Q3 | 12 -- 19 | 13 -- 18 | 12 -- 19 |
| min - max | 6.6 -- 28 | 12 -- 40 | 6.6 -- 40 |
| **Incidence of diseases of the digestive system with respect to patients** |  |  |  |
| N | 19 | 5 | 24 |
| Nmiss | 7 (27%) | 9 (64%) | 16 (40%) |
| mean | 0.13 | 0.11 | 0.12 |
| sd | 0.03 | 0.026 | 0.029 |
| median | 0.13 | 0.12 | 0.12 |
| Q1 - Q3 | 0.1 -- 0.15 | 0.088 -- 0.13 | 0.099 -- 0.14 |
| min - max | 0.081 -- 0.19 | 0.081 -- 0.14 | 0.081 -- 0.19 |
| **Incidence of diseases of the digestive system with respect to occupancy** |  |  |  |
| N | 19 | 5 | 24 |
| Nmiss | 7 (27%) | 9 (64%) | 16 (40%) |
| mean | 0.07 | 0.089 | 0.074 |
| sd | 0.025 | 0.033 | 0.027 |
| median | 0.066 | 0.092 | 0.073 |
| Q1 - Q3 | 0.048 -- 0.087 | 0.058 -- 0.11 | 0.051 -- 0.091 |
| min - max | 0.037 -- 0.12 | 0.051 -- 0.13 | 0.037 -- 0.13 |

**A.2** Original R Output of the fitted models and diagnostics

In all models, the KS test, the Dispersion test and the Outlier test were not conspicuous, except for the last model. Here, the KS test was significant, however the derivation from the expected distribution is marginal.

**A.2.1 Negative binominal model fitted on the EHR data**

Function call:

library(glmmTMB)

m1 <-(glmmTMB(morb_gastro ~ adult + male + n_pat10+

ar1(timef + 0 | type_of_centre),

disp = ~ type_of_centre,

ziformula= ~ n_pat10,

family = nbinom2,

data = EHR_data))

library(DHARMa)

simulateResiduals(fittedModel = m1, plot = T, n=1000)

Output:

Family: nbinom2 ( log )
 Formula:
 morb_gastro ~ adult + male + n_pat10 +
 ar1(timef + 0 | type_of_centre)
 Zero inflation: ~n_pat10
 Dispersion: ~type_of_centre
 Data: EHR_data

 AIC BIC logLik deviance df.resid
 3143.9 3184.9 -1561.9 3123.9 435

 Random effects:

 Conditional model:
 Groups Name Variance Std.Dev. Corr
 type_of_centre timef1 0.03246 0.1802 0.92 (ar1)
 Number of obs: 445, groups: type_of_centre, 2

 Conditional model:
 Estimate Std. Error z value Pr(>|z|)
 (Intercept) 1.266510 0.292522 4.330 1.49e-05 ***
 adult 0.032494 0.037342 0.870 0.384200
 male 0.116248 0.035147 3.308 0.000941 ***
 n_pat10 0.031218 0.001442 21.643 < 2e-16 ***
 ---
 Signif. codes: 0 '***' 0.001 '**' 0.01 '*' 0.05 '.' 0.1 ' ' 1

 Zero-inflation model:
 Estimate Std. Error z value Pr(>|z|)
 (Intercept) -0.81423 0.27401 -2.971 0.00296 **
 n_pat10 -0.07586 0.01911 -3.969 7.21e-05 ***
 ---
 Signif. codes: 0 '***' 0.001 '**' 0.01 '*' 0.05 '.' 0.1 ' ' 1

 Dispersion model:
 Estimate Std. Error z value Pr(>|z|)
 (Intercept) 1.133 0.107 10.592 < 2e-16 ***
 type_of_centreREG 1.953 0.295 6.619 3.61e-11 ***
 ---
 Signif. codes: 0 '***' 0.001 '**' 0.01 '*' 0.05 '.' 0.1 ' ' 1


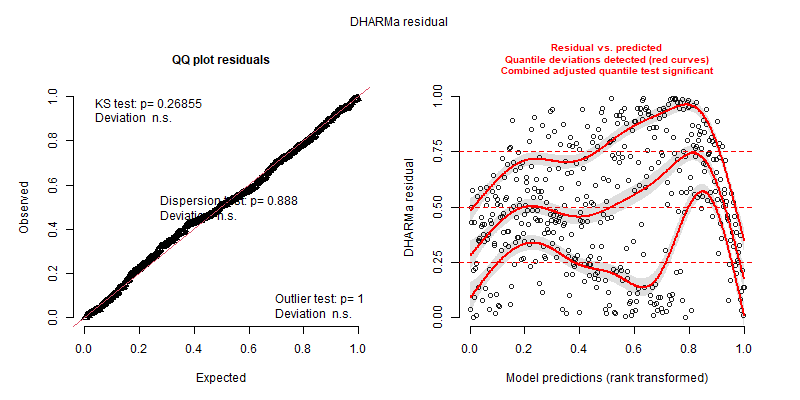


**A.2.2 Generalized linear model fitted on the matched data (occup🡪pat)**

Family: gaussian ( identity )
 Formula:
 n_pat/n_occup ~ adult + male + type_of_centre + n_occup10

 Data: matched

 AIC BIC logLik deviance df.resid
 -141.3 -123.4 76.7 -153.3 141

 Dispersion estimate for gaussian family (sigma^2): 0.0206

Conditional model:
 Estimate Std. Error z value Pr(>|z|)
 (Intercept) 0.0088381 0.0990268 0.089 0.929
 adult 0.0787860 0.0192666 4.089 4.33e-05 ***
 male -0.0096682 0.0147467 -0.656 0.512
 type_of_centreREG 0.3209882 0.0335482 9.568 < 2e-16 ***
 n_occup10 -0.0027022 0.0004974 -5.432 5.56e-08 ***
 ---
 Signif. codes: 0 '***' 0.001 '**' 0.01 '*' 0.05 '.' 0.1 ' ' 1


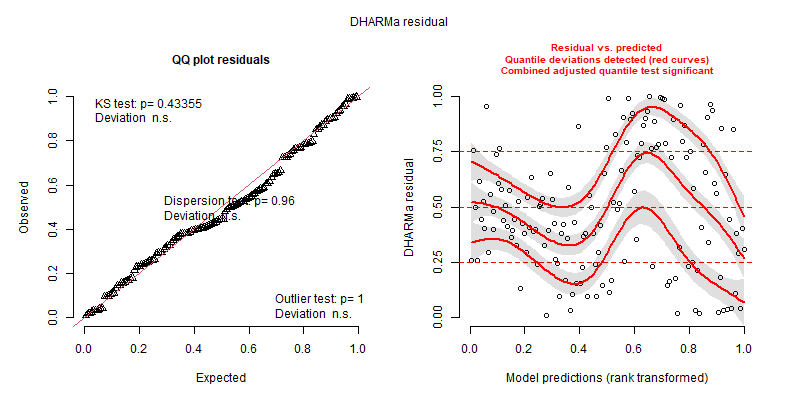


**A.2.3 Generalized linear model fitted on the matched data (pat🡪occup)**

Family: gaussian ( identity )
 Formula:
 n_pat/n_occup ~ adult + male + type_of_centre
 Dispersion: ~n_pat10
 Data: matched

 AIC BIC logLik deviance df.resid
 -92.6 -74.7 52.3 -104.6 141


 Conditional model:
 Estimate Std. Error z value Pr(>|z|)
 (Intercept) 0.36771 0.11811 3.113 0.00185 **
 adult -0.01574 0.02035 -0.773 0.43933
 male 0.04022 0.01561 2.576 0.00999 **
 type_of_centreREG 0.20201 0.03299 6.123 9.15e-10 ***

 ---
 Signif. codes: 0 '***' 0.001 '**' 0.01 '*' 0.05 '.' 0.1 ' ' 1

 Dispersion model:
 Estimate Std. Error z value Pr(>|z|)
 (Intercept) -3.352002 0.169660 -19.757 <2e-16 ***

n_pat10 -0.009298 0.005800 -1.603 0.109
 ---
 Signif. codes: 0 '***' 0.001 '**' 0.01 '*' 0.05 '.' 0.1 ' ' 1


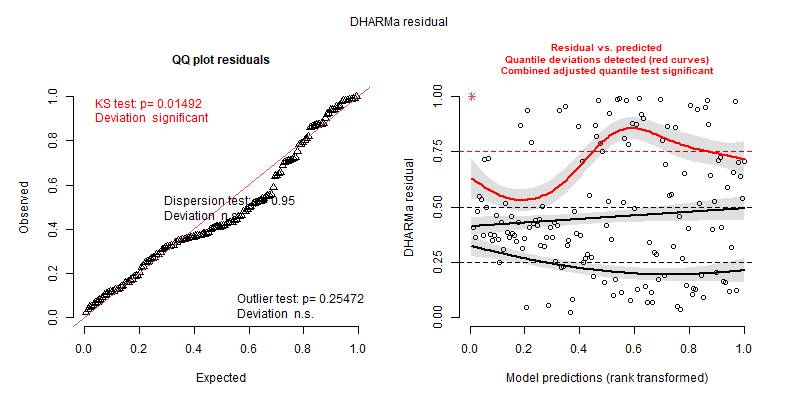


**A.3 Results of sensitivity analysis**

**A.3.1 Sensitivity analysis not excluding** $\mathbf{n}_{\mathbf{occup}}\mathbf{<}\mathbf{n}_{\mathbf{pat}}$

Table A6 is a description of the full matched data, i.e., where records for which the occupancy number is smaller than the number of patients (i.e. $n_{\mathrm{occup}}<n_{\mathrm{pat}}$) were not excluded. It can be seen, that 175-147=28 observations have a ratio greater than one and that the interquartile range and the range of ratios in the full matched data set is given by 0.41 -- 0.81 and 0.13 -- 3.5, respectively. The individual values are given by:

1.02, 1.03, 1.05, 1.05, 1.06, 1.07, 1.08, 1.08, 1.12, 1.12, 1.13, 1.15, 1.17, 1.18, 1.19, 1.24, 1.27, 1.31, 1.33, 1.36, 1.43, 1.97, 2.20, 2.25, 2.36, 3.05, 3.27, 3.47.

Table A7 and A8 show the results of the models described in Sections 2.3.2 and 2.3.3 fitted on this data set. The results are similar with exception of the effect of type of centre. However, this could be explained by the fact, that the mean ratio in the group of REG facilities (=0.99) is biased upwards due to a few very high unrealistic ratios.

**Table A6: Description of full matched data.** Absolute and relative frequencies and mean, standard deviation, median and interquartile range (Q1 - Q3), minimum and maximum of the observed variables of the matched data set stratified by the type of centre are given, respectively. Records for which the occupancy number is smaller than the number of patients (i.e. $n_{\mathrm{occup}}<n_{\mathrm{pat}}$) were not excluded.

| Variables | REC | REG | Total |
| --- | --- | --- | --- |
|  | (N=111) | (N=64) | (N=175) |
| **Month** |  |  |  |
| 2017 11 | 0 (0%) | 0 (0%) | 0 (0%) |
| 2017 12 | 0 (0%) | 0 (0%) | 0 (0%) |
| 2018 01 | 0 (0%) | 0 (0%) | 0 (0%) |
| 2018 02 | 0 (0%) | 0 (0%) | 0 (0%) |
| 2018 03 | 0 (0%) | 0 (0%) | 0 (0%) |
| 2018 04 | 0 (0%) | 0 (0%) | 0 (0%) |
| 2018 05 | 0 (0%) | 0 (0%) | 0 (0%) |
| 2018 06 | 0 (0%) | 0 (0%) | 0 (0%) |
| 2018 07 | 0 (0%) | 0 (0%) | 0 (0%) |
| 2018 08 | 0 (0%) | 0 (0%) | 0 (0%) |
| 2018 09 | 0 (0%) | 0 (0%) | 0 (0%) |
| 2018 10 | 2 (2%) | 1 (2%) | 3 (2%) |
| 2018 11 | 1 (1%) | 0 (0%) | 1 (1%) |
| 2018 12 | 4 (4%) | 2 (3%) | 6 (3%) |
| 2019 01 | 5 (5%) | 2 (3%) | 7 (4%) |
| 2019 02 | 3 (3%) | 1 (2%) | 4 (2%) |
| 2019 03 | 6 (5%) | 2 (3%) | 8 (5%) |
| 2019 04 | 2 (2%) | 0 (0%) | 2 (1%) |
| 2019 05 | 4 (4%) | 1 (2%) | 5 (3%) |
| 2019 06 | 5 (5%) | 2 (3%) | 7 (4%) |
| 2019 07 | 4 (4%) | 1 (2%) | 5 (3%) |
| 2019 08 | 5 (5%) | 2 (3%) | 7 (4%) |
| 2019 09 | 5 (5%) | 2 (3%) | 7 (4%) |
| 2019 10 | 6 (5%) | 2 (3%) | 8 (5%) |
| 2019 11 | 6 (5%) | 1 (2%) | 7 (4%) |
| 2019 12 | 5 (5%) | 2 (3%) | 7 (4%) |
| 2020 01 | 4 (4%) | 2 (3%) | 6 (3%) |
| 2020 02 | 5 (5%) | 3 (5%) | 8 (5%) |
| 2020 03 | 2 (2%) | 1 (2%) | 3 (2%) |
| 2020 04 | 3 (3%) | 3 (5%) | 6 (3%) |
| 2020 05 | 3 (3%) | 3 (5%) | 6 (3%) |
| 2020 06 | 4 (4%) | 3 (5%) | 7 (4%) |
| 2020 07 | 4 (4%) | 1 (2%) | 5 (3%) |
| 2020 08 | 0 (0%) | 1 (2%) | 1 (1%) |
| 2020 09 | 3 (3%) | 1 (2%) | 4 (2%) |
| 2020 10 | 1 (1%) | 1 (2%) | 2 (1%) |
| 2020 11 | 4 (4%) | 2 (3%) | 6 (3%) |
| 2020 12 | 0 (0%) | 1 (2%) | 1 (1%) |
| 2021 01 | 3 (3%) | 2 (3%) | 5 (3%) |
| 2021 02 | 0 (0%) | 3 (5%) | 3 (2%) |
| 2021 03 | 4 (4%) | 4 (6%) | 8 (5%) |
| 2021 04 | 2 (2%) | 4 (6%) | 6 (3%) |
| 2021 05 | 3 (3%) | 4 (6%) | 7 (4%) |
| 2021 06 | 2 (2%) | 4 (6%) | 6 (3%) |
| 2021 07 | 1 (1%) | 0 (0%) | 1 (1%) |
| **Centre** |  |  |  |
| 1 | 3 (3%) | 0 (0%) | 3 (2%) |
| 2 | 0 (0%) | 5 (8%) | 5 (3%) |
| 4 | 16 (14%) | 0 (0%) | 16 (9%) |
| 5 | 13 (12%) | 0 (0%) | 13 (7%) |
| 6 | 17 (15%) | 0 (0%) | 17 (10%) |
| 7 | 0 (0%) | 30 (47%) | 30 (17%) |
| 8 | 1 (1%) | 0 (0%) | 1 (1%) |
| 9 | 0 (0%) | 13 (20%) | 13 (7%) |
| 10 | 7 (6%) | 0 (0%) | 7 (4%) |
| 11 | 13 (12%) | 0 (0%) | 13 (7%) |
| 12 | 0 (0%) | 0 (0%) | 0 (0%) |
| 13 | 0 (0%) | 11 (17%) | 11 (6%) |
| 14 | 9 (8%) | 0 (0%) | 9 (5%) |
| 15 | 11 (10%) | 0 (0%) | 11 (6%) |
| 17 | 16 (14%) | 0 (0%) | 16 (9%) |
| 18 | 4 (4%) | 0 (0%) | 4 (2%) |
| 19 | 1 (1%) | 0 (0%) | 1 (1%) |
| 20 | 0 (0%) | 0 (0%) | 0 (0%) |
| 21 | 0 (0%) | 5 (8%) | 5 (3%) |
| 22 | 0 (0%) | 0 (0%) | 0 (0%) |
| 23 | 0 (0%) | 0 (0%) | 0 (0%) |
| **Male (patients)** |  |  |  |
| N | 111 | 64 | 175 |
| mean | 61 | 61 | 61 |
| sd | 15 | 7.7 | 13 |
| median | 61 | 58 | 59 |
| Q1 - Q3 | 50 -- 71 | 55 -- 67 | 54 -- 70 |
| min - max | 23 -- 90 | 47 -- 79 | 23 -- 90 |
| **Adult (patients)** |  |  |  |
| N | 111 | 64 | 175 |
| mean | 78 | 83 | 80 |
| sd | 11 | 5.6 | 9.8 |
| median | 78 | 84 | 81 |
| Q1 - Q3 | 70 -- 86 | 79 -- 87 | 73 -- 87 |
| min - max | 48 -- 100 | 71 -- 94 | 48 -- 100 |
| **Diseases of the digestive system** |  |  |  |
| N | 111 | 64 | 175 |
| mean | 9.6 | 43 | 22 |
| sd | 10 | 22 | 22 |
| median | 6 | 44 | 12 |
| Q1 - Q3 | 3 -- 13 | 24 -- 62 | 5 -- 33 |
| min - max | 0 -- 45 | 5 -- 83 | 0 -- 83 |
| **n_pat** |  |  |  |
| N | 111 | 64 | 175 |
| mean | 121 | 409 | 226 |
| sd | 55 | 209 | 193 |
| median | 108 | 403 | 143 |
| Q1 - Q3 | 81 -- 148 | 209 -- 590 | 100 -- 269 |
| min - max | 29 -- 274 | 101 -- 934 | 29 -- 934 |
| **Incidence of diseases of the digestive system with respect to patients** |  |  |  |
| N | 111 | 64 | 175 |
| mean | 0.077 | 0.11 | 0.087 |
| sd | 0.058 | 0.026 | 0.05 |
| median | 0.073 | 0.11 | 0.091 |
| Q1 - Q3 | 0.033 -- 0.12 | 0.091 -- 0.12 | 0.055 -- 0.12 |
| min - max | 0 -- 0.23 | 0.05 -- 0.17 | 0 -- 0.23 |
| **Male (occupancy)** |  |  |  |
| N | 111 | 64 | 175 |
| mean | 66 | 65 | 66 |
| sd | 15 | 7.4 | 13 |
| median | 67 | 63 | 66 |
| Q1 - Q3 | 51 -- 78 | 61 -- 70 | 59 -- 75 |
| min - max | 35 -- 94 | 49 -- 97 | 35 -- 97 |
| **Adult (occupancy)** |  |  |  |
| N | 111 | 64 | 175 |
| mean | 78 | 75 | 77 |
| sd | 11 | 8.6 | 10 |
| median | 79 | 75 | 77 |
| Q1 - Q3 | 69 -- 87 | 71 -- 79 | 70 -- 84 |
| min - max | 54 -- 100 | 55 -- 114 | 54 -- 114 |
| **n_occup** |  |  |  |
| N | 111 | 64 | 175 |
| mean | 261 | 553 | 368 |
| sd | 142 | 392 | 297 |
| median | 229 | 417 | 259 |
| Q1 - Q3 | 161 -- 290 | 236 -- 874 | 169 -- 475 |
| min - max | 32 -- 600 | 72 -- 1516 | 32 -- 1516 |
| **Incidence of diseases of the digestive system with respect to occupancy** |  |  |  |
| N | 111 | 64 | 175 |
| mean | 0.036 | 0.11 | 0.062 |
| sd | 0.031 | 0.09 | 0.069 |
| median | 0.031 | 0.079 | 0.049 |
| Q1 - Q3 | 0.016 -- 0.049 | 0.065 -- 0.1 | 0.026 -- 0.079 |
| min - max | 0 -- 0.14 | 0.037 -- 0.46 | 0 -- 0.46 |
| **ratio** |  |  |  |
| N | 111 | 64 | 175 |
| mean | 0.54 | 0.99 | 0.7 |
| sd | 0.27 | 0.65 | 0.5 |
| median | 0.44 | 0.78 | 0.54 |
| Q1 - Q3 | 0.38 -- 0.66 | 0.63 -- 1.1 | 0.41 -- 0.81 |
| min - max | 0.13 -- 2 | 0.37 -- 3.5 | 0.13 -- 3.5 |

**Table A7: Results of the generalized linear model fitted on the full matched data (relationship 2: patient-occupancy ratio when occupancy data is given).** Estimates of the fixed effects of the conditional and dispersion model are given with associated 95% confidence intervals (CI). The number of observations is 175, the AIC is given by 160 and the dispersion estimate for gaussian family is given by 0.136.

| Model | Variable | Estimate | Lower 95% CI | Upper 95% CI |
| --- | --- | --- | --- | --- |
| Conditional | Intercept | 0.51 | 0.05 | 0.96 |
|  | adult (10 %) | -0.03 | -0.11 | 0.06 |
|  | male (10 %) | 0.07 | 0.01 | 0.14 |
|  | Type of centre (ref = REC) | 0.71 | 0.58 | 0.84 |
|  | $n_{\mathrm{occup}}/10$ | -0.01 | -0.01 | -0.01 |

**Table A8: Results of the generalized linear model fitted on the full matched data (relationship 3: patient-occupancy ratio when patient data is given).** Estimates of the fixed effects of the conditional and dispersion model are given with associated 95% confidence intervals (CI). The number of observations is 175, the AIC is given by 194.

| Model | Variable | Estimate | Lower 95% CI | Upper 95% CI |
| --- | --- | --- | --- | --- |
| Conditional | Intercept | 0.42 | -0.02 | 0.86 |
|  | adult (10 %) | -0.06 | -0.14 | 0.02 |
|  | male (10 %) | 0.10 | 0.04 | 0.16 |
|  | Type of centre (ref = REC) | 0.54 | 0.40 | 0.68 |
| Dispersion | Intercept | -2.49 | -2.94 | -2.03 |
|  | $n_{\mathrm{pat}}/10$ | 0.03 | 0.01 | 0.05 |

**A.3.2 Sensitivity analysis excluding observations with 0 cases of diseases of the digestive system**

Due to the exclusion of observations with 0 cases of diseases of the digestive system, the number of observations in the EHR data reduced to 390 (i.e. 55 observations were excluded). Therefore, the number of observations in the matched data set is also reduced (from 147 to 130). Table A9, A10 and A11 show the results of the models of Section 2.3 fitted on the reduced data sets, where the zero-inflation part of the model of Section 2.3.1 was dropped.

In case of “significant” estimates, i.e., 95% confidence intervals of the estimates in the main paper do not contain the “no-effect value” (i.e. they do not contain 1 and 0 for the negative binominal model and for the generalized linear models, respectively), the results of the main analysis resemble that of the sensitivity analysis.

**Table A9: Results of the negative binominal model fitted on the reduced EHR data (relationship1: disease incidence).** Estimates of the fixed effects of the conditional and dispersion model are given with associated 95% confidence intervals (CI). The number of observations is 390, the AIC is given by 2850.0 and the first-order autoregressive coefficient is given by 0.92.

| Model | Variable | incident rate ratio | Lower 95% CI | Upper 95% CI |
| --- | --- | --- | --- | --- |
| Conditional | Intercept | 3.64 | 2.09 | 6.35 |
|  | adult (10 %) | 1.03 | 0.96 | 1.11 |
|  | male (10 %) | 1.12 | 1.05 | 1.20 |
|  | $n_{\mathrm{pat}}/10$ | 1.03 | 1.03 | 1.03 |
| Dispersion | Intercept | 3.24 | 2.66 | 3.95 |
|  | type of centre (ref = REC) | 6.79 | 3.83 | 12.04 |

**Table A10: Results of the generalized linear model fitted on the reduced matched data (relationship 2: patient-occupancy ratio when occupancy data is given).** Estimates of the fixed effects of the conditional and dispersion model are given with associated 95% confidence intervals (CI). The number of observations is 130, the AIC is given by -137.9 and the dispersion estimate for the gaussian family is 0.0185.

| Model | Variable | Estimate | Lower 95% CI | Upper 95% CI |
| --- | --- | --- | --- | --- |
| Conditional | Intercept | 0.11 | -0.08 | 0.31 |
|  | adult (10 %) | 0.08 | 0.04 | 0.11 |
|  | male (10 %) | -0.03 | -0.05 | 0 |
|  | type of centre (ref = REC) | 0.34 | 0.27 | 0.40 |
|  | $n_{\mathrm{occup}}/10$ | 0 | 0 | 0 |

**Table A11: Results of the generalized linear model fitted on the reduced matched data (relationship 3: patient-occupancy ratio when patient data is given).** Estimates of the fixed effects of the conditional and dispersion model are given with associated 95% confidence intervals (CI). The number of observations is 130, the AIC is given by -93.0.

| Model | Variable | Estimate | Lower 95% CI | Upper 95% CI |
| --- | --- | --- | --- | --- |
| Conditional | Intercept | 0.51 | 0.27 | 0.76 |
|  | adult (10 %) | -0.03 | -0.07 | 0.02 |
|  | male (10 %) | 0.03 | -0.01 | 0.06 |
|  | type of centre (ref = REC) | 0.23 | 0.17 | 0.30 |
| Dispersion | Intercept | -3.43 | -3.77 | -3.08 |
|  | $n_{\mathrm{pat}}/10$ | -0.01 | -0.02 | 0.00 |

**A.3.3 Sensitivity analysis including country of origin**

As information on country of origin of patients were available in the EHR data, but not in occupancy data, we performed a sensitivity analysis. We selected the five most frequent countries of origin in our data. These countries were: Nigeria, Afghanistan, Syria, Iraq and Turkey. For each of these countries we calculated the percentage of patients with this country of origin per month and centre. We then performed a combination of forward and backward selection procedure (1) with respect to the AIC for the models of relationship 1 and 3.

**Table A12: Results of the negative binominal model fitted on the EHR data including countries of origin (relationship1: disease incidence).** Estimates of the fixed effects of the conditional, zero-inflation and dispersion model are given with associated 95% confidence intervals (CI). The number of observations is 445, the Akaike information criterion (AIC) is given by 3107.7 and the first-order autoregressive coefficient is given by 0.80.

| Model | Variable | incidence rate ratio (IRR) | Lower 95% CI | Upper 95% CI |
| --- | --- | --- | --- | --- |
| Conditional | Intercept | 1.69 | 0.89 | 3.19 |
|  | adult (10 %) | 1.14 | 1.05 | 1.23 |
|  | male (10 %) | 1.12 | 1.05 | 1.19 |
|  | $n_{\mathrm{pat}}/10$ | 1.04 | 1.03 | 1.04 |
|  | Nigerian patients (10 %) | 0.9 | 0.85 | 0.95 |
|  | Afghani patients (10 %) | 1.06 | 1.01 | 1.12 |
|  | Syrian patients (10 %) | 0.93 | 0.88 | 0.98 |
|  | Iraqi patients (10 %) | 0.9 | 0.8 | 1.01 |
| Zero-inflation | Intercept | 0.43 | 0.25 | 0.74 |
|  | $n_{\mathrm{pat}}/10$ | 0.93 | 0.89 | 0.96 |
| Dispersion | Intercept | 3.72 | 2.98 | 4.65 |
|  | Type of centre, REG (ref: REC) | 4.92 | 2.85 | 8.49 |

**Table A13: Results of the generalized linear model fitted on the matched data (relationship 3: patient-occupancy ratio when patient data is given).** Estimates of the fixed effects of the conditional and dispersion model are given with associated 95% confidence intervals (CI). The number of observations is 147, the AIC is given by -118.6.

| Model | Variable | Estimate | Lower 95% CI | Upper 95% CI |
| --- | --- | --- | --- | --- |
| Conditional | Intercept | 0.4 | 0.11 | 0.7 |
|  | adult (10 %) | -0.03 | -0.08 | 0.01 |
|  | male (10 %) | 0.03 | 0.01 | 0.06 |
|  | Type of centre. REG (ref: REC) | 0.25 | 0.18 | 0.32 |
|  | Nigerian patients (10 %) | 0.04 | 0.02 | 0.06 |
|  | Afghani patients (10 %) | 0.03 | 0.00 | 0.05 |
|  | Syrian patients (10 %) | 0.03 | -0.01 | 0.07 |
|  | Iraqi patients (10 %) | -0.08 | -0.15 | -0.02 |
|  | Turkish patients (10 %) | 0.11 | 0.04 | 0.18 |
| Dispersion | Intercept | -3.75 | -4.11 | -3.39 |
|  | $n_{\mathrm{pat}}/10$ | 0.00 | -0.02 | 0.01 |

**A.4 Additional figures including 95% confidence intervals**


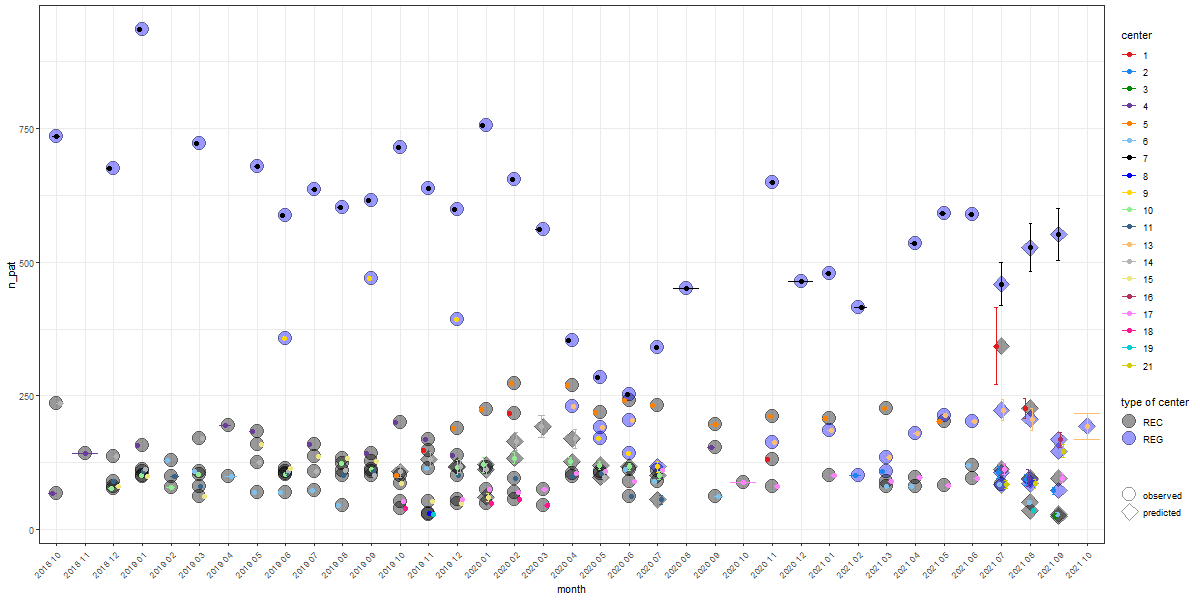


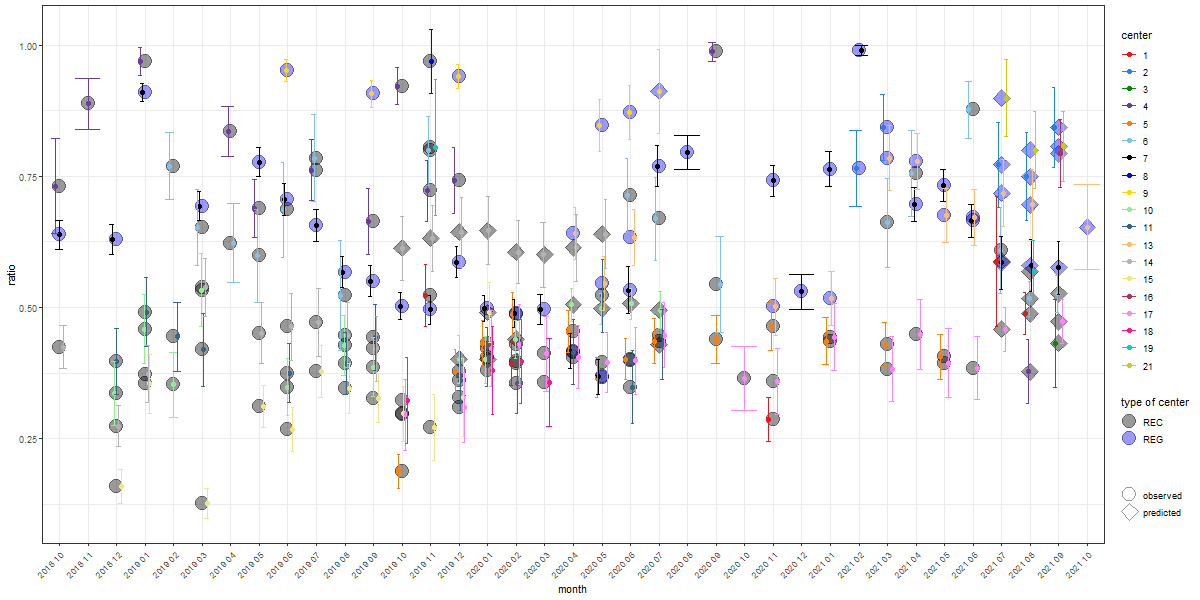


**Figure A1: Observations and predictions on relationship 2: patient-occupancy ratio when occupancy data is given.** Observed (indicated by circles) and predicted (indicated by diamonds) ratio $r$ and number of patients $n_{pat}=n_{pop}\cdot r$ on the basis of the matched data set and the model of Section 2.3.2 for the unmatched occupancy dataset, respectively. Observations/predictions of different facilities are indicated by the colours of the small dots inside the circles/diamonds, which are coloured with respect to the type of centre (REC: black, REG: blue). The associated 95% confidence intervals for the observed estimates/ predictions are indicated by error bars.

**
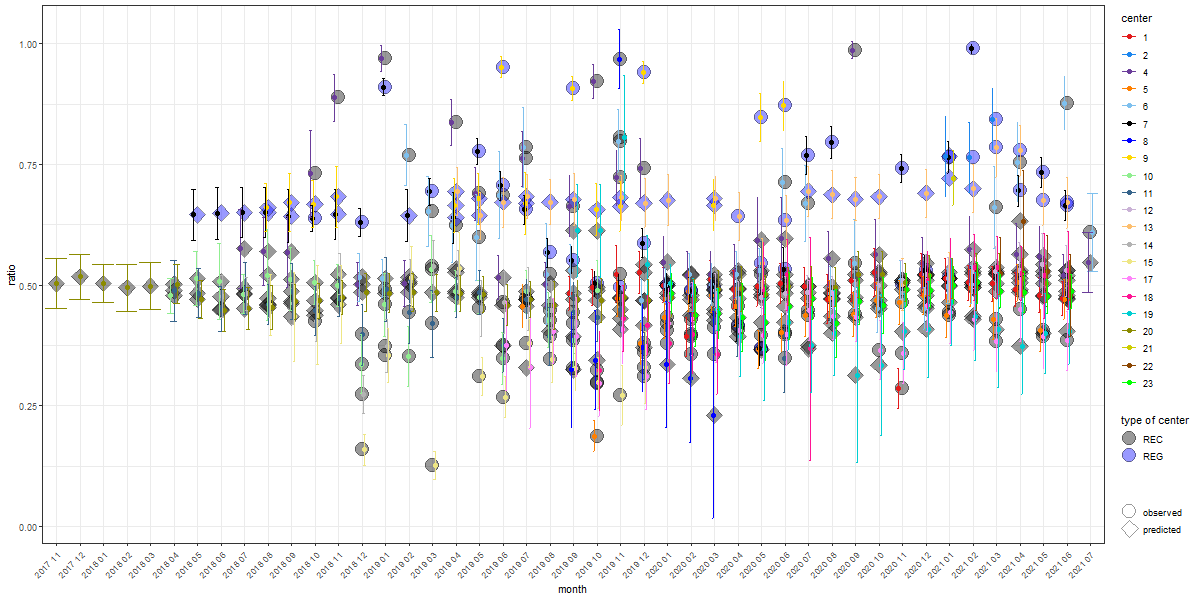
**

**
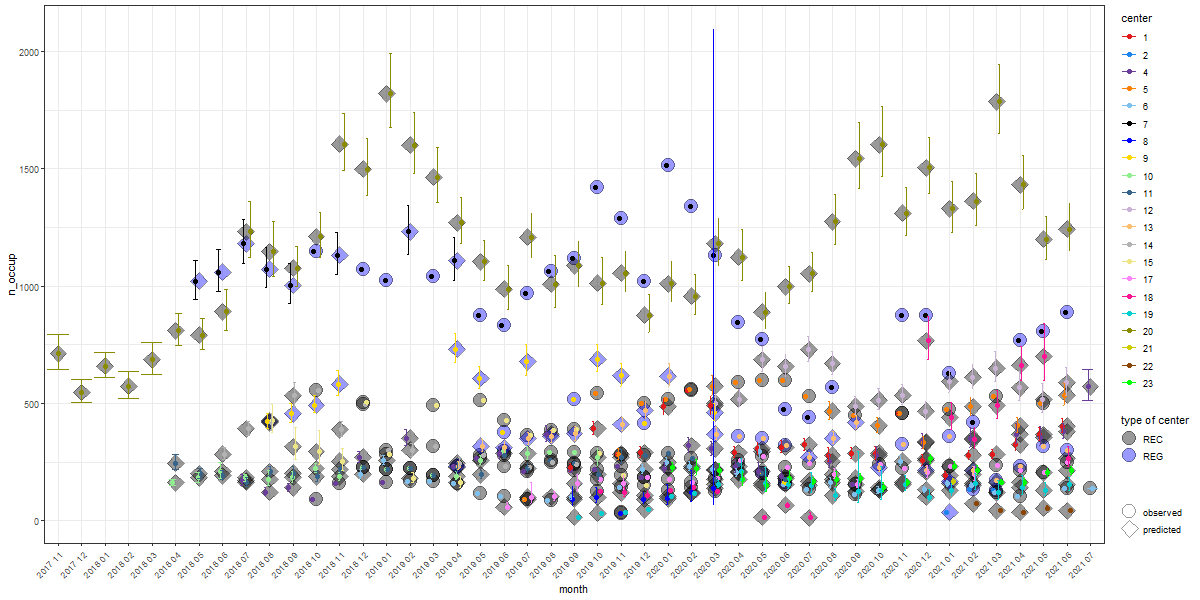
**

**Figure A2: Observations and predictions on relationship 3: patient-occupancy ratio when patient data is given.** Observed (indicated by circles) and predicted (indicated by diamonds) ratio $r$ and number of persons $n_{occup}=n_{pat}/r$ on the basis of the matched data set and the model of Section 2.3.3 for the unmatched EHR dataset, respectively. Observations/predictions of different facilities are indicated by the colours of the small dots inside the circles/diamonds, which are colored with respect to the type of centre (REC: black, REG: blue). The associated 95% confidence intervals for the observed estimates/ predictions are indicated by error bars.


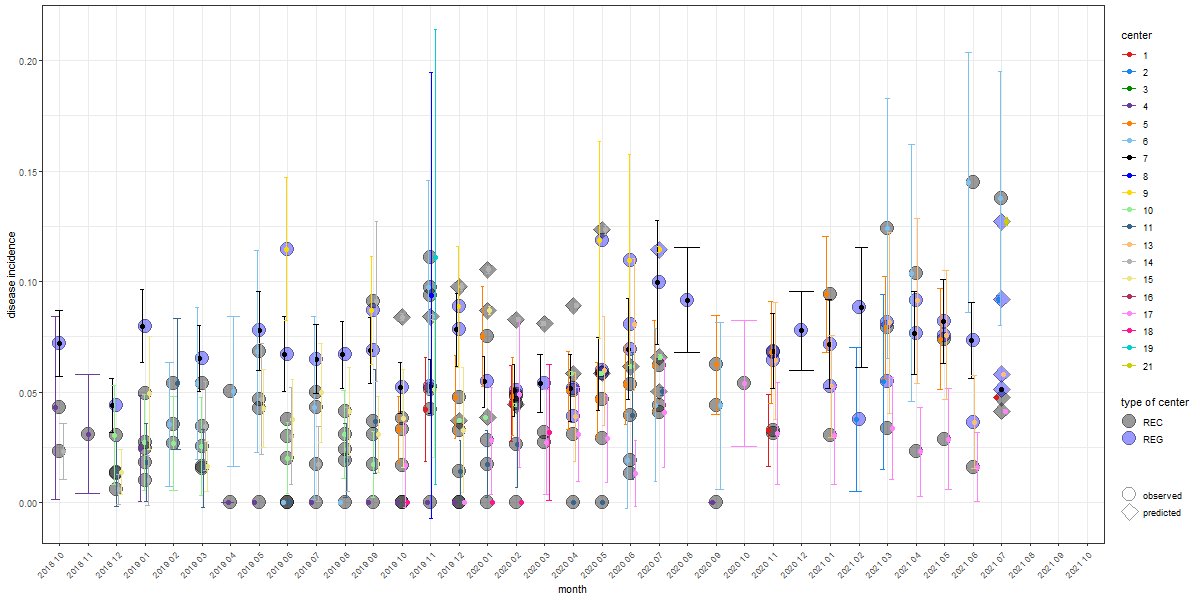


**Figure A3: Observations and predictions on relationship 1 and 2: disease incidence depending on total population at risk (occupancy).** Observed (indicated by circles) and predicted (indicated by diamonds) disease incidence with respect to occupancy number (i.e., number of diseases of the digestive system divided by the occupancy number) on the basis of the EHR data set and the models of Section 2.3.2 (relationship 2: patient-occupancy ratio when occupancy data is given) and Section 2.3.1 (relationship 1: disease incidence) for the unmatched occupancy dataset, respectively. Observations/predictions of different facilities are indicated by the colours of the small dots inside the circles/diamonds, which are coloured with respect to the type of centre (REC: black, REG: blue). The associated 95% confidence intervals for the observed estimates/ predictions are indicated by error bars.

**References**

1. Gabel M, Hohl T, Imle A, Fackler OT, Graw F. FAMoS: A Flexible and dynamic Algorithm for Model Selection to analyse complex systems dynamics. PLOS Computational Biology. 2019;15(8):e1007230.
